# A probabilistic map of motor and cognitive functions: a meta-analysis of 4325 stimulation sites

**DOI:** 10.1007/s00429-025-03015-2

**Published:** 2025-11-12

**Authors:** Anna Alexandratou, Viktoria Sefcikova, Michael S. Elmalem, Jeroen Bisschop, Sibel Emilie Huet, George Samandouras, Parashkev Nachev

**Affiliations:** 1 Neurology Department, Evangelismos Hospital, Athens, Greece; 2 UCL Queen Square Institute of Neurology, University College London, Queen Square, London, UK; 3 The University of Queensland Medical School, Brisbane, Australia; 4 Caboolture Hospital, Caboolture, Australia; 5 High-Dimensional Neurology Group, Department of Translational Neuroscience and Stroke, UCL Queen Square Institute of Neurology, Queen Square, London, UK; 6 Max Planck Institute for Human Cognitive and Brain Sciences, Leipzig, Germany; 7 Institute of Diagnostic and Interventional Radiology, University Hospital Zurich, University of Zurich, Zurich, Switzerland; 8 Department of Neurosurgery, The National Hospital for Neurology and Neurosurgery, Victor Horsley, Queen Square, London, UK; 9 High Dimensional Neurology Group, UCL Queen Square Institute of Neurology, University College London, Russell Square House, Bloomsbury, London, UK

**Keywords:** Direct electrical stimulation, Meta-analysis, Disconnectome, Functionnectome, Neurosurgery, Topological inference

## Abstract

Direct electrical stimulation (DES) is a spatially controlled, clinically informed form of lesion-deficit analysis. This study aims to generate the first meta-analytic, probabilistic cognitive maps based on DES and subdural stimulation data. We systematically searched PubMed, Embase, and Web of Science for studies that used intraoperative DES or subdural electrodes and reported coordinates linked with disrupted performance of a behavioural task. These positive loci and their corresponding intraoperative tasks were collated and grouped into behavioural categories. Data were analysed to determine the relationship between a locus in standard stereotactic space and reported behaviours. Barycentric coordinates, focal maps, and outputs of disconnectome and functionnectomic analysis are provided. We identified 21 eligible studies totalling 954 patients with 4325 positive stimulation sites. Sites were categorized into seven behaviours. Five behaviours yielded significant clusters, including (1) motor with clusters within the pre-central gyri and anterior right cingulate gyrus; (2) somatosensory in the bilateral post-central gyri; (3) auditory comprehension in the left superior temporal gyrus; (4) visuospatial, namely face perception, in the fusiform gyrus; and (5) speech and language, specifically, naming associated with the left posterior superior temporal gyrus, perseveration in left insular cortex and white matter, and speech arrest within precentral gyri. Disconnectome analysis generated 56 significant clusters across seven behaviours. Our meta-analysis generates novel, probabilistic cognitive maps which provide converging evidence for well-established functional-anatomical relationships, as well as areas of ongoing study.

**Supplementary Information:**

The online version contains supplementary material available at 10.1007/s00429-025-03015-2.

## Introduction

Scientific efforts spanning over 150 years have aimed to reveal the relationship between cognitive functions and discrete cortical areas. Among the earliest brain mapping techniques was lesion-deficit analysis, which inferred the function of an anatomical region based on observed deficits following brain damage, and included the seminal work of Paul Broca and Karl Wernicke (Broca 1861; Wernicke 1874). However, the functional topological inferences derived from these early methods vary in reproducibility (Tremblay and Dick 2016; Dronkers et al. 2007). For example, in re-evaluations of the lesions described by Broca using high-resolution MRI, the affected regions were more medial and encompassed a larger volume than originally described (Dronkers et al. 2007).

The use of lesions to infer functional anatomy is impeded by their complex heterogeneity, an inevitable consequence of the nature of the underlying pathological processes (Mah et al. 2014; Xu et al. 2018). Though this has historically compelled a shift to correlative methods, such as functional imaging and voxel-based morphometry, as the dominant approach to mapping brain function, the unique causal power of disruptive methods has motivated efforts to overcome the methodological challenges they present (Rorden and Karnath 2004). This requires attention to two aspects of the inferential process: the representation of the neural substrate, and of lesions themselves. The objective in both cases is to represent variation in a space compact enough to allow efficient, unbiased inference while remaining expansive enough to accommodate fine anatomical detail of functional significance (Giles et al. 2023).

A popular solution to the first task is to discretize the anatomy of the brain into a comparatively small set of putatively homogeneous parcels. This has been traditionally approached through a cytoarchitectonic lens, exemplified by Korbinian Brodmann’s cytoarchitectural map composed of 52 histologically distinct and functionally separate Brodmann Areas (BAs) (Zilles 2018). Over the years, cytoarchitectonic maps have been further refined to capture heterogeneity within specific BAs, amounting to approximately 150–200 parcels (Amunts and Zilles 2015; Blumensath et al. 2013; Zilles and Amunts 2012). However, functional-anatomical associations made on the basis of simple, single histological templates remain insecure, owing to lack of sensitivity to inter-subject variability, absence of a precise coordinate system in stereotaxic space, and no depiction of connectivity through white matter tracts. Myeloarchitectonic maps have in-part addressed these limitations by considering the characteristics of myelinated fibres (Vogt 1910). The lack of real-time feedback was addressed by supplementing myelinated fibre analysis with electrophysiological mapping of non-human primates (Vogt 1910). A modern analysis of the Vogt dataset, aligned to MNI space, produced a total of 180 myeloarchitectonic areas (Nieuwenhuys and Broere 2017). The use of any parcellation scheme implies functional exchangeability of the elements within each parcel: an assumption manifestly violated by the meso- and microscopic heterogeneity of the brain’s anatomy, and unsafe even if homogeneity could be obtained. Structurally identical substrates may perform different functions at different locations, just as biochemically identical neurotransmitters subserve different functions in different pathways. The quest for an optimal representation of the neural substrate remains open.

The challenges of the second task—optimally representing lesions—are mirrored (Xu et al. 2018). To the extent to which any N areas are co-lesioned in a given patient, their differential contributions cannot be ascertained, and to the extent to which any N areas tend to be co-lesioned across the population, the resultant structured pattern of covariance will tend to systematically mislocalize the inferred critical locus. High-dimensional multivariate methods, based on partial implicit or comprehensive explicit representations of lesion distributions, can address this problem, but will always be limited by the structure of the sampling process peculiar to each lesion-generating pathological mechanism (Pustina et al. 2018; Zhang et al. 2014; Pombo et al. 2023).

One solution arising in a neurosurgical context is directed, focal electrical stimulation through either (a) implanted subdural electrodes and extraoperative stimulation, or (b) intraoperative direct electrical stimulation (DES) for the removal of brain tumours in critical areas or epileptogenic foci. Transient intraoperative stimulation of the brain parenchyma creates electrical discharges reversibly incapacitating highly localised, approximately 5 mm^2^, cortical sites or subcortical white matter segments. The stimulated site is considered ‘positive’ when an observed change is noted during execution of a task, compared to pre-stimulation performance. Therefore, the change of behavioural outcome elicited by inhibition of a specific site allows us to infer its function.

While DES offers precise spatial interference, the number and location of stimulation sites are dictated by clinical necessity, rather than an exhaustive exploration of brain regions. This has resulted in a literature with an abundance of single-institution studies with incomplete spatial data. Therefore, constructing maps that incorporate both focal and connectivity effects is crucial. These maps offer a more comprehensive understanding of the broader network-level disruptions caused by stimulation at a single point. Recent efforts have been made to collate and analyse data from this patient group and present illustrative proof of cortical and subcortical distribution of behaviours (Coletta et al. 2024; Elmalem et al. 2023; Lu et al. 2021; Rech et al. 2019; Sarubbo et al. 2020; Puglisi et al. 2019; Fornia et al. 2020b, 2024; Desmurget et al. 2018; Caruana et al. 2016, 2018). Fornia et al. (2024) used postoperative lesion-symptom mapping with support vector regression to identify cortical clusters associated with praxis and prehension deficits, while Puglisi et al. (2019) combined lesion-symptom mapping with preoperative diffusion tractography to reveal white matter tracts underlying cognitive control disruptions identified intraoperatively. However, collating and synthesising positive electrical stimulation sites to generate a novel brain map has yet to be performed.

The objective of the present paper is to generate a functional-anatomical map based on eloquent stimulation sites through a systematic review and meta-analysis of the available literature. At the same time, we also provide functionnectomic analysis (i.e., illustration of the probability of distant sites being disrupted through white matter projections associated with eloquent stimulation sites).

## Methods

### Search strategy and selection criteria

The protocol for this meta-analysis is registered at the International Prospective Register of Systematic Reviews (PROSPERO ID: CRD42022356862; Online Appendix 1.1). A systematic search was performed on PubMed, Embase via Ovid, and Web of Science from database inception to Nov 2, 2022. No language restrictions were applied. The complete search strategy is reported in Online Appendix 1.2. The entries were imported into EndNote X9 for de-duplication. To deem eligible, studies had to include primary research available in English and published in peer-reviewed journals with acceptable study design including case studies, clinical trials, cohort studies, case reports, and/or case-control. Studies had to report electrical stimulation through implanted subdural electrodes or a monopolar or bipolar probe intraoperatively on patients undergoing neurosurgery, as well as reports of clear numerical MNI or Talairach coordinates for positive sites. A ‘positive site’ was defined as a site that either disrupted, annulled, or increased an already present behaviour upon stimulation. Corresponding authors and investigators were contacted to supplement incomplete reports on coordinates of positive sites.

Titles and abstracts were independently screened by two reviewers (AA and JB). Articles deemed potentially relevant by at least one reviewer were retrieved for full text review. Risk of bias was evaluated using the NIH Quality Assessment Tool for Case Series Studies, which was modified to remove “adequate length of follow up” as it was not applicable. The eloquent stimulation points were independently extracted by two authors (AA, VS). To facilitate comparisons between studies, eloquent sites were reported in MNI space by translating Talairach coordinates using the Bioimage Talairach to MNI converter from the Yale BioImage Suite Package (Papademetris et al. 2006). Coordinates were independently categorised according to behaviour altered or neutralised upon stimulation by two reviewers (AA, VS). Inconsistencies in functional categorisation were resolved by consensus. The anatomical site of the cortical and subcortical barycentric locations were based on the Harvard-Oxford Brain Atlas and John Hopkins University (JHU) White Matter Tractography Atlas (Mori et al. 2005).

### Data analysis

In this study, three distinct sets of analyses were performed. The first focused on deriving cortical maps of focal grey matter regions associated with a specific behavioural deficit. The second involved a disconnectome analysis, which projected the cortical stimulation contact points onto white matter tracts using tractography-based priors. The third analysis extended the scope by using functionnectomic priors to identify connected grey matter regions across the brain, applying the same inferential framework introduced by Elmalem and colleagues (Elmalem et al. 2023).

### Local disruptive mapping

Stimulation locations and their associated behavioural subcategories were extracted. Each location was represented as a volume (1 × 1 × 1 mm sampled), with an intensity of zero at all but the stimulated location, where the intensity was one. The behaviour-specific MNI stimulation locations are shown in the supplementary Fig. 2.9, found in the Online Appendix.

Then, each image was convolved with a 3D Gaussian kernel of 10 mm full-width-half-maximum (FWHM), enabling the modelling of spatial uncertainty in the spatial location of the contact point and approximation of the focal propagation of the induced disruption (Elmalem et al. 2023). The resultant image, therefore, contained a single Gaussian blob centred at the reported meta-analytic location, capturing spatial uncertainty as the density of the spatial distribution across all voxels in the volume. Spatial density was defined as the normalized number of stimulation points per voxel, based on a 2 mm MNI152 space. Importantly, this method enables the group analysis of sparse clinical data, as it considers variations between subjects and variations in functional-anatomical relationships that are not accounted for by anatomical registrations, similar to the methodology applied in meta-analytic modeling of functional activation data (Eickhoff et al. 2009, 2012), and recently extended within the framework of statistical parametric mapping (SPM) (Engleitner et al. 2022).

Having derived a map for each location, a sum image was generated and masked by applying a threshold where electrode density was > 0.0001 to exclude poorly sampled areas (i.e., areas with extremely limited stimulation coverage, which would be unlikely to generate reliable or interpretable results). For each behavioural category, the smoothed stimulation images were entered into a voxel-wise general linear model with electrode density as the dependent variable and the binary behavioural effect as the independent variable. For each of the behavioural models, a one-sample t-test was conducted at each voxel. To account for multiple comparisons, the resultant t-maps were thresholded at *p* < 0.05 FWE (peak voxel).

### Disconnectomic disruptive mapping

Given the set of smoothed stimulation coordinates, we used BCBtoolkit Disconnectome module to reconstruct brain connectivity maps, weighed on white matter effects, depicting disconnectomic probability from a stimulation seed point and the white matter tracts it is likely to disrupt (Foulon et al. 2018). In brief, as extensively described elsewhere, this approach uses a set of 178 healthy participants diffusion-weighted imaging (DWI) datasets to track fibres passing through each lesion. For each of the 178 participants tractography was estimated as described elsewhere (Thiebaut de Schotten et al. 2011). Patients’ lesions in MNI152 space were registered to each control native space using affine and diffeomorphic deformations and subsequently used as seed for tractography analysis in Trackvis (trackvis.org) (Klein et al. 2009; Avants et al. 2011; Wang et al. 2007).

Tractographies from the lesions were transformed into visitation maps, binarised and registered to the MNI152 using the inverse of precedent deformations (Thiebaut de Schotten et al. 2011). Finally, percentage overlap maps were generated by summing at each point in MNI space the normalised visitation map of each healthy subject. Hence, in the resulting disconnectome map, the value in each voxel takes into account the interindividual variability of tract reconstructions in controls and indicates a probability of disconnection from 0 to 100% for a given lesion (Thiebaut de Schotten et al. 2015). Specifically, each stimulation point was dilated to obtain a 3D binary sphere with an isotropic diameter of 5 voxels, resulting in a binary, lesion-like volume. We then fed the resulting dilated stimulations to the disconnectome module, to obtain a continuous probabilistic disconnection map.

As the resulting maps are already dense (in contrast with the sparser focal disruption maps), for the disconnectomic analysis we applied a smaller smoothing kernel of 6 mm FWHM prior to assessing the association for each behavioural outcome with the same statistical design as used for local disruption mapping. Again, a voxel-wise repeated-measures general linear model with the binary behavioural effect as a factor was used. One sample voxel-wise t-tests were performed and thresholded at *p* < 0.05 FWE (peak voxel), now revealing disconnectome maps. We sought to visualise the effect magnitudes, therefore we thresholded the beta maps at the corresponding critical p-value and then normalised the resulting maps between 0 and 1.

The resulting inferential disconnectomic maps represent statistically significant white matter tracts associated with the set of lesions for each of the behavioural models. We sought to extend the analysis to account for grey matter effects, now representing the critically connected grey matter voxels that are statistically likely to be disrupted given a stimulation location, now treated as a functional signal. We, therefore, performed a third and final set of analyses––functionnectomic disruptive mapping––as described below.

### Functionnectomic disruptive mapping

We used the functionnectome software (http://www.bcblab.com and https://github.com/NotaCS/Functionnectome) to generate functional white matter maps that are likely to be functionally disconnected given a grey matter activation locus, here modeled as a Gaussian. In short, the functionnectome relies on anatomical priors derived from the tractography of 100 healthy participants from the Human Connectome Project (HCP). These priors consist of voxel-specific probabilities of structural connectivity with all other brain voxels, totalling in 228,453 maps (Nozais et al. 2021).

As the software requires 2 mm isotropic resolution 4D volumes as input, each smoothed location was converted to a 4D functional image, containing two identical frames. Whole-brain white matter probabilistic priors were used to generate a functionnectomic map for each of the images, resulting in a map showing the functional white matter tracts and their grey matter destinations that are likely to be disrupted given the grey matter signal embedded in the input image. The same modeling procedures used in the disconnectomic case were applied once again—now revealing functionnectome maps.

### Visualisation

Visualisation was done using the SurfIce toolbox (https://www.nitrc.org/projects/surfice*)* for focal and functionnectome maps. We used FSL’s HCP1065 standard-space FA atlas to generate a background FA template converted into a mesh using SurfIce’s volume-to-mesh function and overlaid each NIfTI file to the template. Lateral left and right hemisphere views were taken for the focal maps for each behaviour surviving the threshold. Lateral, superior and inferior views were taken for the functionnectome maps for each behaviour. The grey matter anatomical labels were determined by using the aal atlas, which is a built-in atlas to the MRIcroGL software (Tzourio-Mazoyer et al. 2002). The normalised disconnectome maps were visualised using MRIcroGL (Chris Rorden, v.12.5) at the FWE corrected threshold in different axial views. For white matter locations, the natbrainlab in-built atlas was utilised, as well as JHU atlas for each coordinate, for creation of accurate anatomical labels. The nearest grey matter location to the maxima of each statistical cluster was determined using SPM12’s neuromorphometrics grey matter atlas for cortical regions. The figures were designed on Inkscape (https://inkscape.org).

## Results

The search yielded 11,971 results. After deduplication, 6015 remained for abstract screening, and full-text retrieval took place for 886 articles. A total of 21 studies met all eligibility criteria and were included in analysis (Online Appendix 2.1) (Mani et al. 2008; Roux et al. 2009, 2012, 2014, 2015; Matsumoto et al. 2011; Tate et al. 2014; Wu et al. 2015; Rech et al. 2016; Herbet et al. 2017a, b; Chang et al. 2017; Zacà et al. 2018; Yordanova et al. 2019; Mandonnet et al. 2019; Sarubbo et al. 2020; Schrouff et al. 2020; Simone et al. 2021; Zhou et al. 2021; Ng et al. 2021; Giampiccolo et al. 2022).

All included studies were determined to have low risk of bias (Online Appendix 2.2). The risk of bias was generally low for every domain within each trial, with the exception of the majority of studies not being consecutive series. There was one study with population not clearly described (Mani et al. 2008); the same study also lacked clear description of statistical methods employed. Two more studies fall behind a clear description of their statistical methods, as well (Matsumoto et al. 2011; Rech et al. 2016).

A total of 954 patients were identified across the 21 included studies (Online Appendix 2.3). Sex was reported in 20 studies totaling 43.4% female patients (*n* = 413) and 56.6% male (*n* = 538). The weighted average age was 40.4 years. Patient handedness was documented in 20 studies, further specified as right in 89.5% (*n* = 761) of patients, left in 8% (*n* = 68), and ambidextrous in 2.5% (*n* = 21). In three studies, totaling 104 patients, handedness was either not reported (Chang et al. 2017) or incomplete (Matsumoto et al. 2011; Schrouff et al. 2020). Brain mapping involved intraoperative DES in 18 included studies (*n* = 941 patients, 98.6% of the total cohort) and extraoperative stimulation of subdural electrodes in three studies (*n* = 13, 1.4%) (Mani et al. 2008; Matsumoto et al. 2011; Schrouff et al. 2020). In the studies employing DES, the pathological specimen was low-grade glioma (LGG) in 82.4% of patients, high-grade glioma (HGG) in 5.6%, cavernous angioma in 0.3%, and the remaining 11.7% were other/non-specified. For subdural electrodes, all patients had refractory epilepsy.

The specific intraoperative tasks used for each study are described in Online Appendix 2.4. Similar stimulation parameters were used to elicit motor responses across included studies—a handheld bipolar probe of either 5–6 mm spacing with biphasic wave current of 1 msec at 60 Hz. One study deviated from these methods, including high frequency stimulation (250 Hz) (Simone et al. 2021). For amplitude, the majority of studies applied 1–8 mA for any given behaviour (Roux et al. 2009, 2012, 2014, 2015; Tate et al. 2014; Wu et al. 2015; Rech et al. 2016; Chang et al. 2017; Herbet et al. 2017a, b; Zacà et al. 2018; Mandonnet et al. 2019; Yordanova et al. 2019; Sarubbo et al. 2020; Ng et al. 2021; Simone et al. 2021; Zhou et al. 2021; Giampiccolo et al. 2022), Whereas studies using implanted electrodes used higher currents, reaching 15 mA (Mani et al. 2008; Matsumoto et al. 2011). Amplitude was not reported in one study (Schrouff et al. 2020).

Data extraction identified 4325 eloquent stimulation sites, categorized into one of seven general behavioural categories: motor, somatosensory, speech and language, non-verbal semantics, auditory comprehension, mentalizing, and visuospatial. Sub-categories were generated based on intraoperative tasks. Barycentre coordinates and locations are provided in Online Appendix 2.5.

### Motor

Overall, 1090 eloquent sites across seven studies were associated with motor behaviour (Roux et al. 2009; Tate et al. 2014; Chang et al. 2017; Sarubbo et al. 2020; Simone et al. 2021; Rech et al. 2016; Zhou et al. 2021). Two motor subcategories were generated: (1) movement, documented in five studies, included the majority of sites (*n* = 816 sites, 75%) (Roux et al. 2009; Tate et al. 2014; Chang et al. 2017; Sarubbo et al. 2020; Simone et al. 2021); and (2) motor control, reported in three studies, contained the remaining sites (*n* = 274) (Rech et al. 2016; Sarubbo et al. 2020; Zhou et al. 2021). Eloquent sites were evenly distributed between the hemispheres. For movement, the barycentre was located within the pre-central gyrus, bilaterally, with spread including the precentral, postcentral, superior frontal, and middle frontal gyri (MFG). For motor control, barycentres were in the right post-central and left MFG with anatomical distribution involving the precentral gyrus, pre-motor cortices, supplementary motor area (SMA), pre-SMA, insula, basal ganglia, caudate and neighbouring matter, and the posterior part of the middle frontal gyrus.

### Somatosensory

For somatosensation, 356 eloquent sites were collated from three studies with the majority of reported sites collected from the right hemisphere (*n* = 222, 62%) (Tate et al. 2014; Chang et al. 2017; Sarubbo et al. 2020). Bilaterally, the barycentre was located in the post-central gyrus, while the anatomical spread additionally included the thalamocortical tracts.

### Speech and language

A total of 2261 eloquent sites were identified for speech and language of which 88% (*n* = 1848) were located in the left hemisphere. Articulation was its own sub-category with three aspects—speech arrest, verbal apraxia, and perseveration—analysed separately. The remaining sub-categories were phonology, reading, naming, neologistic, and verbal semantics.

Coordinates associated with speech arrest, including anarthria and dysarthria, were reported by six studies totalling 1128 eloquent sites located predominantly in the left hemisphere (*n* = 963; 85%) (Tate et al. 2014; Chang et al. 2017; Sarubbo et al. 2020; Wu et al. 2015; Zhou et al. 2021; Zacà et al. 2018). Bilaterally, the barycentre was within the precentral gyrus and the site distribution further included the premotor cortex and underlying white matter, inferior postcentral gyrus, pars opercularis (POp), and superior temporal gyrus (STG).

Verbal apraxia was recorded in one study totalling 184 sites with barycentres in the precentral gyri, bilaterally (Sarubbo et al. 2020). Further areas of spread included the inferior and middle frontal gyri, pre- and post-central gyri, STG, supramarginal gyrus and its underlying white matter, including part of the superior longitudinal fasciculus.

Perseveration was documented in two studies with 47 total sites predominantly in the left hemisphere (98%) where the barycentre was located within the anterior limb of the internal capsule and putamen and anatomical distribution was primarily subcortical, surrounding the basal ganglia and within its neighbouring white matter (Mandonnet et al. 2019; Sarubbo et al. 2020). The barycentre of the right hemisphere is based on a single eloquent site in the anterior limb of the internal capsule.

Eloquent sites associated with phonology were recorded in four studies amounting to 156 sites, which were left-lateralized (*n* = 147; 94%) (Roux et al. 2014; Tate et al. 2014; Sarubbo et al. 2020; Ng et al. 2021). The barycentre in the left and right hemispheres were the central opercular cortex (POp) and insular cortex, respectively. The anatomical spread in the left hemisphere involved the underlying white matter connecting the premotor cortices, located in the posterior frontal lobe, and various cortical regions, namely the inferior and middle frontal gyri, and the posterior two-thirds of the temporal lobe, with all three temporal gyri involved.

A total of 152 eloquent sites were reported for reading across six studies (Roux et al. 2009, 2012, 2014; Ng et al. 2021; Mani et al. 2008; Sarubbo et al. 2020). Of these, 98.7% were within the left hemisphere with the posterior STG as the barycentre. The anatomical distribution included the middle temporal gyrus (MTG), inferior temporal gyrus (ITG), basal and lateral white matter associated with the ITG, and the fusiform gyrus, namely the dorsal aspect of the inferior longitudinal fasciculus (ILF).

Naming was disrupted in eight studies yielding 582 eloquent sites, 97% of which were in the left hemisphere (Roux et al. 2009, 2014, 2015; Tate et al. 2014; Wu et al. 2015; Chang et al. 2017; Sarubbo et al. 2020; Ng et al. 2021). Barycentric coordinates were in the central (POp) and parietal opercular cortex in the left and right hemisphere, respectively. Frontal site distribution included the inferior frontal gyrus (IFG), namely the POp and pars triangularis (PTri), as well as the posterior two-thirds of the STG and MTG. Sites were also detected at the inferior parietal lobule, notably the angular gyrus.

Twelve sites associated with neologistic jargonaphasia were documented in one study (Giampiccolo et al. 2022). All sites were situated in the left hemisphere with the barycentric average coordinate in Heschl’s gyrus with eloquent sites clustered along the arcuate fasciculus (AF).

A total of 170 eloquent sites were documented in association with verbal semantics across four studies (Tate et al. 2014; Giampiccolo et al. 2022; Sarubbo et al. 2020; Ng et al. 2021). The majority were in the left hemisphere (*n* = 162; 95%) with the left and right barycentres in the insular cortex. Cortically, eloquent sites were scattered along the STG, MTG, supramarginal gyrus (SMG), dorsal premotor cortex, and IFG. Subcortically, sites spanned white matter fibres of the ILF and inferior frontal occipital fasciculus (IFOF) within the deep temporal lobe, ascending to the IFG and MFG.

### Non-verbal semantics

Eloquent sites for non-verbal semantics were captured by three studies (Herbet et al. 2017a; Roux et al. 2015; Sarubbo et al. 2020). This amounted to 90 points, which were evenly distributed between the hemispheres (right = 48.9%, left = 51.1%). Bilaterally, the barycentre was in the central opercular cortex (POp). In contrast to verbal semantics, the non-verbal counterpart had sites spanning the STG, middle third of the MTG, IFG, and dorsolateral prefrontal cortex (DLPFC).

### Auditory comprehension

Auditory comprehension was documented in three studies (Matsumoto et al. 2011; Roux et al. 2015; Sarubbo et al. 2020). A total of 115 sites included reports of acoustic interference and sound recognition. Eloquent sites were left-lateralized (99.1%) with a barycentre in the posterior part of the STG. In addition to the site concentration in the STG, and the white matter underneath, more posterior sites were located in the SMG and MTG. Few sites were found at the POp.

#### Mentalizing

Mentalizing disruption was captured by two studies with 41 sites total, all located within the right hemisphere with a barycentre in the MFG (Sarubbo et al. 2020; Yordanova et al. 2019). There was distribution over the IFG, frontal white matter, DLPFC, and all three components of the IFG, the POp, PTri, and pars orbitalis.

### Visuospatial

For visuospatial behaviour, 202 eloquent sites were extracted, with an almost equal distribution in the right (*n* = 102, 50.5%) and the left (*n* = 100, 49.5%) hemisphere. Subcategories were created for spatial perception, vision, face perception, and writing.

Spatial perception was described in three studies with 58 eloquent sites (Sarubbo et al. 2020; Tate et al. 2014; Herbet et al. 2017b). The majority (87.9%) of sites were in the right hemisphere with a barycentre in the parietal opercular cortex, whereas the barycentre in the left was the SMG. In the right, anatomical distribution involved the SMG and underlying white matter, posterior STG, MTG, MFG, and the white matter of the frontal lobe following the course of the superior longitudinal fasciculus (SLF).

A total of 43 eloquent sites for vision were collated from two studies (Sarubbo et al. 2020; Schrouff et al. 2020). Right barycentre, computed with 58.1% of these sites, was in the right optic radiation, whilst the barycentre in the left hemisphere was found in left optic radiation.

Face perception was captured in one study using subdural electrodes in which a total of 27 stimulation sites were reported in pairs (Schrouff et al. 2020). The sites were right-lateralised (*n* = 25, 92.6%) and the barycentre was found in the fusiform gyrus.

Writing was documented in two studies with the distribution of 74 eloquent sites heavily left-lateralised (98.3%) (Roux et al. 2009, 2014). The barycentre was in the anterior division of the SMG, while further sites were found in the STG.

**Fig. 1:**
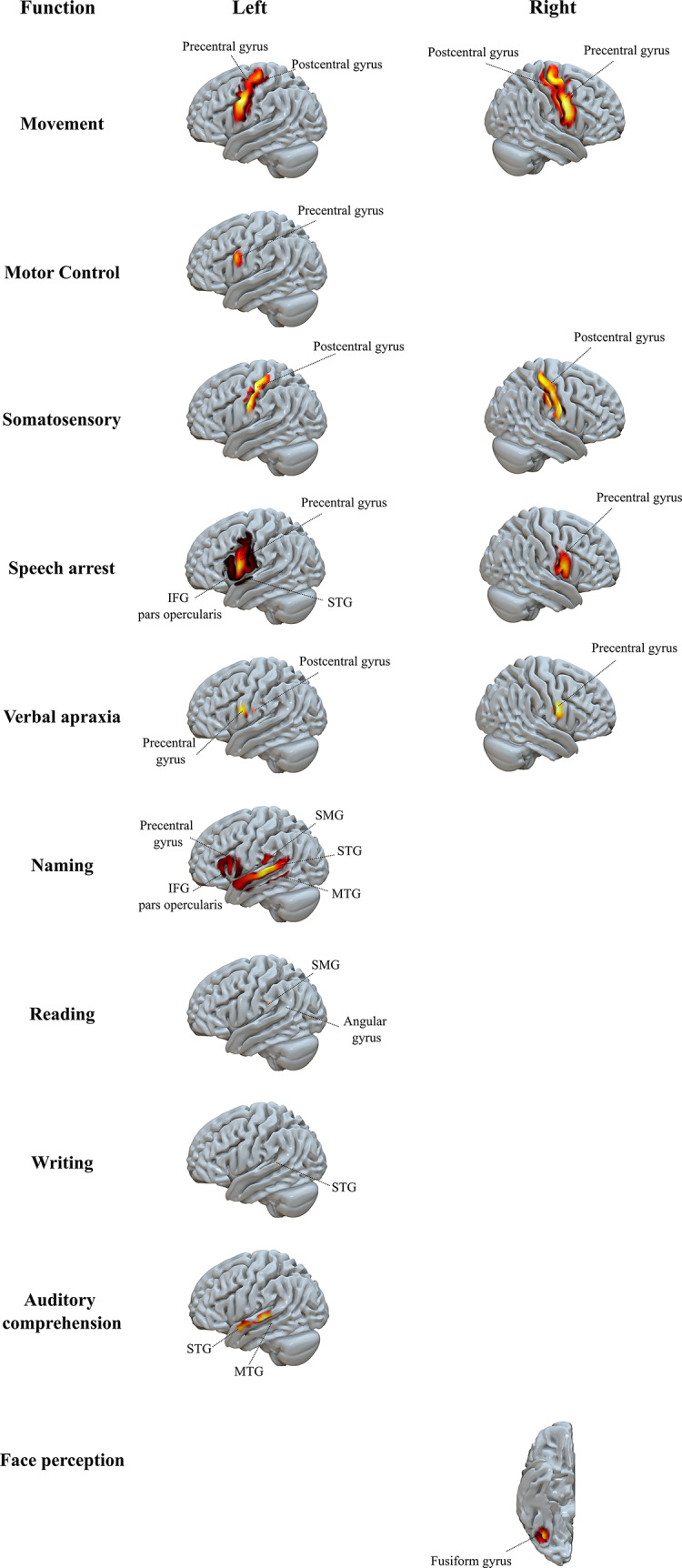
Focal maps of eloquent stimulation sites

Five of the seven general behavioural categories yielded statistically significant (FWE corrected *p* < 0.05) eloquent stimulation clusters (Online Appendix 2.6). See Fig. 1 for the associated maps including behavioural subcategories. Specifically, there were six significant clusters for movement in the bilateral precentral gyri, two for motor control located in the left precentral gyrus and right anterior division of the cingulate gyrus, six clusters for somatosensory behaviour within the bilateral postcentral gyri, four for speech arrest each in the precentral gyrus, three for naming in the posterior division of the STG, three for auditory comprehension in the anterior and posterior division of the STG, three for perseveration in the white matter and insular cortex, and one cluster was generate for face perception, located in the fusiform gyrus.

**Fig. 2:**
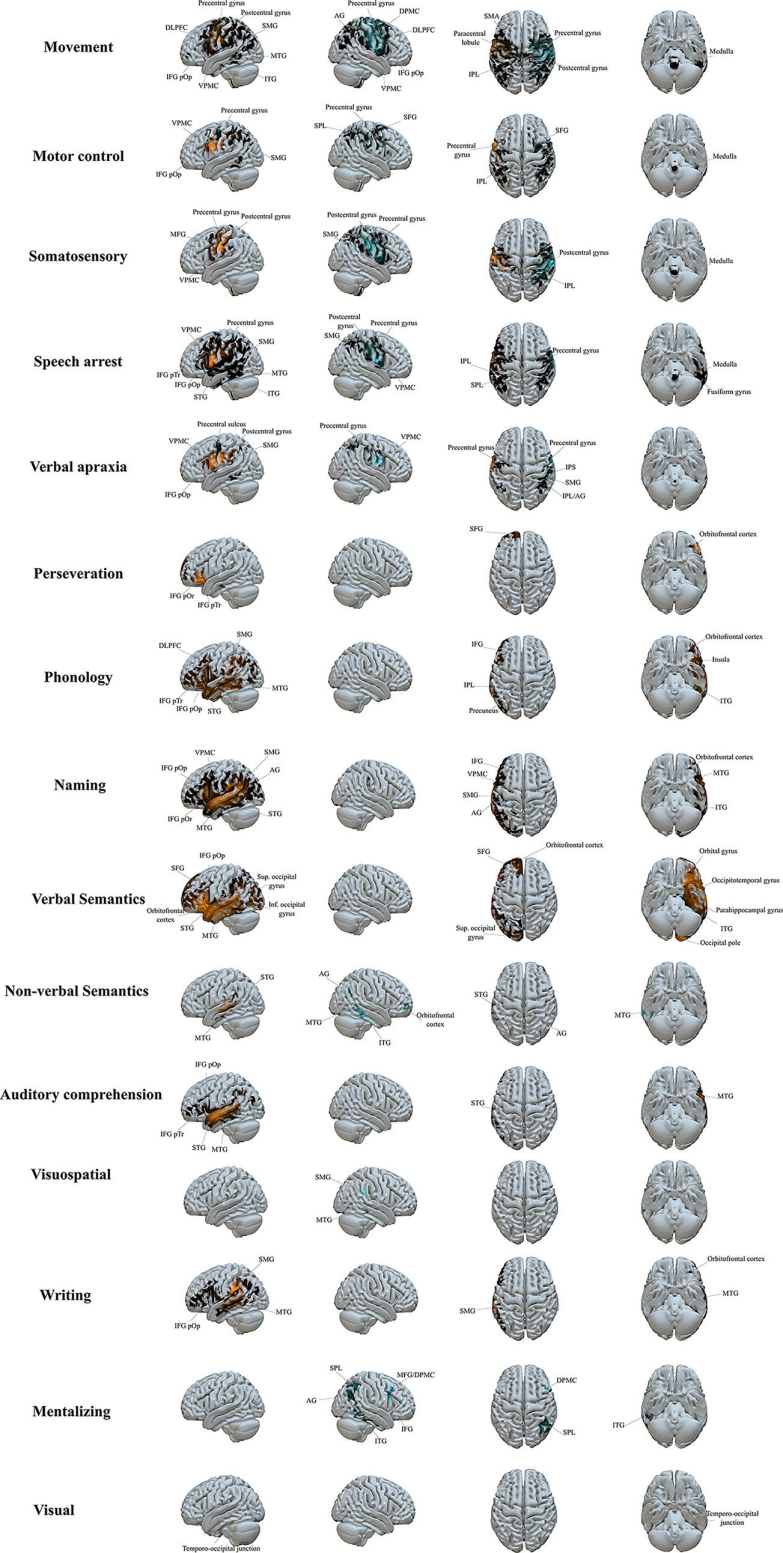
Functionnectome maps depicting critically connected grey matter voxels statistically likely to be disrupted from a stimulation location. For each MNI voxel, beta contrast was performed. Only voxels surviving the *p* < 0.05 FEW-corrected threshold are shown, with higher beta values, depicted in brighter colors, representing a stronger association between the stimulation sites and the behaviour

Functionnectome maps were created for the seven behaviours, separated into their respective subcategories where relevant (Fig. 2). See Online Appendix 2.7 for the MNI coordinates and anatomical location associated with each disconnectome cluster. The disconnectome maps are provided in Online Appendix 2.8.

## Discussion

DES is a widely accepted brain mapping technique with clinical applications dating back to the late 19th century. As it is the only tool that induces transient disruption with stimulation radius estimates of only a few mm^2^, DES is the gold standard for brain mapping, allowing for highly localized, real-time testing of cognitive behaviours. The literature on functional-anatomical mapping lacks meta-analyses integrating large-scale outputs from DES or subdural stimulation. The present, clinically-validated meta-analytic map allows for inferences of causality between (a) the observed changes in cognition and (b) the stimulated region and applied current.

The present maps model the underlying neural basis of the tested cognitive behaviours with high congruency between the anatomical sites identified in the literature and our maps, in particular for sites associated with reading (Zemmoura et al. 2015; Shin et al. 2019), spatial perception (Marshall et al. 2002; Herweg and Kahana 2018), auditory comprehension (Liebenthal et al. 2003; Schönwiesner et al. 2007), mentalizing (Salzman and Fusi 2010; Schurz et al. 2014; Dixon et al. 2017; Laurita et al. 2017), and face perception (Kanwisher et al. 1997). We also support motor control as a multi-centric function.

Our findings support movement initiation is not confined to areas traditionally described as part of the motor cortex. Rather, as identified in our focal, disconnectome, and functionnectome maps, it further involves regions of the post-central gyrus or primary somatosensory cortex (S1), which has a similar somatotopic arrangement as the pre-central gyrus (i.e., primary motor cortex; M1) (Penfield and Rasmussen 1950; Geyer et al. 2000). Our movement findings attributed to S1 stimulation align with prior reports (Fang et al. 2020; Geyer et al. 2000). Anatomically, motor activity within S1 could be explained by the presence of U-shaped fibres linking the M1 and S1 gyri (Sanderson et al. 1984). U-shaped fibres are short-range association fibres that connect two gyri in close proximity through the adjacent sulci—in this case, the central sulcus. Using tractography and post-mortem dissection, Catani and colleagues found the hand region of S1 to possess the largest volume of U-fibres, which gradually decrease in volume for other areas of S1 (Catani et al. 2012). Similarly, Fornia and colleagues report the presence of U-fibres between the post-central and pre-central gyri, again emphasizing the abundance of this network in the motor and sensory area of the hand (Fornia et al. 2020a; Viganò et al. 2019). The large portion of direct connections between the hand-knob area and S1 could provide useful feedback to facilitate skilled hand motor abilities, including precision grasping and finger coordination (Simone et al. 2021; Thompson et al. 2017).

In the context of U-fibres, two hypotheses could explain the presence of positive movement sites upon DES stimulation of discrete regions of S1: (1) trans-synaptic spread of DES current from M1 to S1 through U-fibres, which is supported by the lower threshold of activation of M1 compared to S1; or (2) the elimination of inhibition, in which the U-fibres allow for the tonic inhibition of S1 by M1 and the DES current eliminates this inhibition, allowing S1 to regulate and control M1 via corticospinal fibres.

However, other hypotheses have been proposed for S1’s association with movement—of which includes functional compensation of the postcentral gyrus in the presence of precentral gliomas (Bennett et al. 2022). Alternatively, studies in primates show anterior parietal regions, including S1 areas 3a and 2, contain corticospinal neurons capable of directly influencing spinal motor circuits (Strick et al. 2021). It has been shown in macaques that stimulation of S1, particularly area 1, could elicit hand movements even during reversible deactivation of M1, a potential independent motor contribution from parietal cortex (Bresee et al. 2024).

For somatosensation, while S1 contained expected significant voxels, the functionnectome depicts voxels extending into the bilateral precentral gyri, as well as the right anterior supramarginal gyrus. The right supramarginal gyrus has been shown to activate during proprioceptive processing (Ben-Shabat et al. 2015). Co-activation occurs in premotor areas, overlapping with or near the precentral gyrus, suggesting these areas are involved in the integration of proprioceptive signals into motor planning (Ben-Shabat et al. 2015).

While there is substantial variability surrounding the anatomical regions ascribed ‘Broca’s Area’ across studies, typical definitions include either the pTri and pOp exclusively or the IFG in its entirety (Tremblay and Dick 2016). Our focal cortical maps of grey matter regions associated with three types of articulatory disturbances—speech arrest, verbal apraxia, and perseveration— did not identify statistically significant clusters in Broca’s Area. Instead, statistically significant foci for speech arrest were concentrated within the left precentral gyrus. Within the pre-central gyrus, the ventral premotor cortex is a centre of motor planning and coordination consistently related to speech output (Catani et al. 2012; Seghier et al. 2014; Meister et al. 2007). Although verbal apraxia and perseveration did not yield significant clusters in our focal maps, disconnectome analysis associated verbal apraxia with the arcuate fasciculus, SLF-I, and SLF-II. The arcuate fasciculus has been associated with bidirectional mapping of speech articulation and acoustics—connecting phonological schemes to motor sequences allowing for repetition (Catani et al. 2005; Saur et al. 2008). The SLF-I may be related to control of higher aspects of motor function and initiating articulatory activity, although its fibres have high potential to be misattributed to the cingulum (Wang et al. 2016). In contrast, SLF- II, has robust support for its involvement in the motor aspects of speech processing (van Geemen et al. 2014). Findings of articulation being related to the superior thalamic radiations and corticospinal tract reflect expected engagement of motor output pathways (Stockert et al. 2025; Matchin and Hickok 2020).

Our findings suggest that DES of Broca’s area alone may less frequently disrupt articulation in isolation; instead, its proximity to articulatory motor regions, such as the inferior precentral gyrus, supports an integrative role in organizing speech output. This aligns with contemporary models of speech production, which postulate Broca’s area contributes to higher-order planning and sequencing, while the ventral precentral gyrus executes articulatory movements (Hickok and Poeppel 2007; Tremblay and Dick 2016). Our probabilistic maps confirm Broca’s area involvement, particularly the pars opercularis, in speech arrest, reinforcing its clinical importance during intraoperative monitoring and refining classical views by situating it within a distributed fronto-temporal articulatory network (Amunts et al. 1999; Dronkers et al. 2007; Indefrey and Levelt 2004).

Interestingly, significant clusters for speech arrest were also identified in the STG. While the STG is traditionally linked with semantic and lexical processing (Lu et al. 2021), speech arrest sites have been reported in this region, highlighting substantial interindividual variability in speech arrest localization (Chang et al. 2017; Zacà et al. 2018). Lu et al. (2021) found that although speech arrest sites most commonly localize to the inferior frontal gyrus and ventral precentral gyrus, a smaller number were observed in the posterior STG. Authors suggest these may reflect disruption of feedback-based articulatory monitoring mechanisms, rather than direct motor output (Lu et al. 2021). Given the inconsistencies in the literature and the possibility of stimulation current spread and anatomical variability, these findings warrant cautious interpretation. There is a need for future studies employing high-density and surface-based mapping approaches for precise delineation of speech articulation areas.

Our analysis revealed significant clusters for speech arrest in the right pre-central gyrus, as well. Right frontal activation during speech production has been observed in healthy patients, often linked to fine-tuning of responses to improve efficiency rather than altering accuracy (Hartwigsen et al. 2010). While the present findings could be alternatively related to right-hemispheric speech dominance (Knecht et al. 2000), there has been growing evidence supporting the association between right-hemispheric lesions and speech and language deficits, supporting the relevance of our right-hemisphere findings (Gajardo-Vidal et al. 2018; Yamamoto et al. 2022).

Picture naming remains among the most commonly used tasks for intraoperative mapping. Our data support that picture naming engages extensive cortical networks—beginning with the visual cortex, which then projects the signal to regions that eventually assign meaning to visual stimuli, and finally, phonological retrieval to name the visual object aloud. Anomia is a deficit characterized by inability to name visually presented objects, and, as such, it can result from a disturbance in phonological and/or semantic processing (Duffau et al. 2014). This complexity is reflected in our disconnectome results, which implicated the middle longitudinal fasciculus, IFOF, optic and acoustic radiations, and forceps minor—structures contributing to both ventral and dorsal language streams (Sefcikova et al. 2022). The middle longitudinal fasciculus and specific temporo-frontal pathways within the ventral language stream are critical for semantic processing. In particular, connections linking the intermediate lateral temporal cortex to the lateral prefrontal cortex via the temporofrontal extreme capsule fasciculus (TFexcF) have been shown to support lexical and semantic retrieval, as highlighted by Barbeau et al. (2024). This temporo-frontal segment is distinct from more posterior occipital-frontal fibers of the IFOF and reflects the core anatomical substrate of the ventral semantic network, consistent with the integral roles of both the TFexcF and IFOF in language comprehension and production (Barbeau et al. 2024).

Interestingly, although the AF is classically considered a phonological pathway, but it was not detected in our disconnectome analysis under naming, but rather non-verbal semantics. As early as 1972, Geschwind suggested that a more dorsal pathway involving the AF promotes speech repetition and conceptually driven speech production, such as object naming (Geschwind 1972). Our findings supports models of the AF which describe an ‘indirect segment’ connecting temporal and parietal regions, hypothesized to be involved in semantic processing as described by Catani and colleagues, and later supported in DTI work by Glasser and Rilling (Catani et al. 2005; Glasser and Rilling 2008). While naming-related semantic processes rely primarily on ventral pathways including the uncinate fasciculus, IFOF, and the anterior ILF segment which connects the anterior and posterior temporal lobe (Moritz-Gasser et al. 2013; Mehta et al. 2016; Herbet et al. 2016). More recent evidence indicates that such effects might, at least partially, reflect the role of the temporo-frontal extreme capsule fasciculus (Barbeau et al. (2024), which is becoming increasingly acknowledged for playing a seminal role in semantic integration.

We identify an array of limitations of our study.

First, while the largest cohort from our meta-analysis (*n* = 256 patients) from Sarubbo et al. (2020) is also included in a recent lesion network mapping study by Coletta et al. (2024), our work differs in several ways. In contrast to the study by Coletta and colleagues, which performs predictive modeling and seeds DES-generated transient lesions into a normative functional connectome, our study sources DES sites through meta-analysis and builds probabilistic disruption maps directly from stimulation data (Coletta et al. 2024). Our work combines focal mapping, disconnectome mapping, and uniquely functionnectome mapping. Our study also extends analysis to a wider range of cognitive domains. Our findings support the lesion-disconnectome models proposed by Coletta et al. (2024), most notably in terms of the significance of the temporo-frontal white matter tracts in speech production.

Second, some limitations are inherent to the pathologies within the review sample, specifically 82.4% of patients had low-grade gliomas, which due to their slower growth compared to high-grade counterparts, have a propensity to gradually reorganize brain networks to compensate for the loss of the eloquent region. The recruitment of alternative neural networks can be unpredictable, potentially limiting generalisability (Kinoshita et al. 2014; van Dokkum et al. 2019). In contrast, high-grade tumours exhibit rapid growth, meaning less time is available for reorganization, and brain networks are retained to a greater degree. This allows for better localisation of brain functions consistent with pre-morbid functioning.

Third, there is lack of consensus with regard to the choice of sensitive and specific intraoperative mapping tasks. The variability in task selection used to assess a behaviour leads to challenges in interpreting outcomes associated with applied stimulation.

Fourth, several intraoperative tasks have overlapping stages of processing which may be related to deficits, which means certain behaviours such as vision and reading, due to the tasks used, are not mutually exclusive, and specific inputs from reading could add further data points to the vision maps. In our study, separate maps were generated based on the type of intraoperative task employed at the expense of number of stimulation sites used to generate the respective maps.

Fifth, several studies were excluded during the selection process as numerical coordinates were not provided and stimulated sites were instead depicted on a figure. Future studies could interrogate a larger data set by translating these coordinates to MNI space. There would be a trade-off between increased statistical power and increased chance of error/inter-observer variability. Further, the DES literature lacks reports ‘negative’ DES sites (i.e., stimulation sites which do not evoke a behavioural response for a given function). Systematic reporting of null responses would enable direct spatial comparison between regions where stimulation did and did not elicit behavioural effects, improving statistical robustness. Analysis could be further enriched by systematic reporting of types of probes (e.g., bipolar vs. monopolar) and frequency utilised for both positive and negative DES sites.

Sixth, we did not model the intensity of the stimulation at which the behavioural impact was registered, as it is both rarely reported and hard to quantify at the critical tissue level. Stimulation is assumed to disrupt the local substrate, in equal volume and to an equal extent across studies. This is clearly a simplification, but not a plausible source of anatomically organised bias across the sampled areas.

Seventh, no distinction is made between electrically induced neural activation and inhibition of the underlying substrate, for the precise physiological effects are likely to be both complex and locus dependent. However, since the stimulation is profoundly non-physiological, it is reasonable to model it as unsigned interference, in line with the approach pursued in the domain of transcranial magnetic stimulation.

Eighth, we acknowledge that voxel-based approaches, while widely used, may introduce anatomical imprecision due to spatial smoothing and volume-based normalization—particularly across complex sulcal boundaries. Use of smoothing to approximate individual variability could suffer in lack of capturing glioma-related anatomical interruptions, with displacement and reorganization of white matter tracts. Ideally, subject-level tractography would provide a more accurate representation of individual connectivity. Future studies may also benefit from adopting surface-based techniques and geodesic mapping of stimulation sites to reduce blurring and improve anatomical localization accuracy, especially when visualizing stimulation effects in highly convoluted cortical regions (Bellacicca et al. 2024; Avanzini et al. 2016).

Finally, the unavoidable clinical context of direct cortical stimulation involves both pathological and compensatory changes, operating at various temporal scales, that may introduce departures from normal functional anatomy. Obtaining signals marginalized over a diversity of pathologies offers some protection from such distortion, but cannot offer any theoretical guarantees. Equally, the consequences of focal disruption of a network need not replicate its behaviour under distributed disruption: that inevitably requires multi-focal disruption for which limited investigational methods (other than pathological lesions) are available.

In conclusion, this study represents the first attempt at generating a meta-analytic neuroanatomical map based on neurosurgical electrical stimulation sites. DES and subdural stimulation allows for precise, immediate evaluation of various cognitive behaviours in a clinical setting. Further, DES provides a framework for causal inferences between cognitive findings and the specific brain region stimulated, which can be used to generate a new functional-anatomical model of the brain.

## Supplementary Information

Supplementary Material 1

## Data Availability

No datasets were generated or analysed during the current study.
