# Supplementary material for: A probabilistic map of motor and cognitive functions: a meta-analysis of 4325 stimulation sites
Source: Brain Struct Funct. 2025 Nov 12;230(9):178. doi: 10.1007/s00429-025-03015-2 (PMC12611997; doi:10.1007/s00429-025-03015-2)
Supplement: Supplementary file 1 — Supplementary Material 1 [file 429_2025_3015_MOESM1_ESM.docx]

**A probabilistic map of motor and cognitive functions: a meta-analysis based on 4325 stimulation sites**

Anna Alexandratou MD, Viktoria Sefcikova MD, MSc, Michael Elmalem MSc, Jeroen Bisschop MD, Sibel Emilie Huet MD, George Samandouras MD, Parashkev Nachev PhD

Appendix

**TABLE OF CONTENTS**

[1. METHODS 3](#_Toc203414553)

[1.1 Protocol 3](#_Toc203414554)

[1.2 Search strategy 6](#_Toc203414555)

[2. RESULTS 7](#_Toc203414556)

[2.1 PRISMA flow diagram depicting the study selection process 7](#_Toc203414557)

[2.2 Risk of bias table of included studies 8](#_Toc203414558)

[2.4 Intraoperative tasks 10](#_Toc203414559)

[2.4 Behaviours 12](#_Toc203414560)

[2.5 Stimulations sites and Barycentre coordinates associated with each function 13](#_Toc203414561)

[2.6 Statistical values and anatomical location of clusters of different functions 14](#_Toc203414562)

[2.7 Statistical values and anatomical location of disconnectome clusters of different behaviours 15](#_Toc203414563)

[2.8 Disconnectome maps 19](#_Toc203414564)

[2.9 Maps of the distribution of stimulation points 27](#_Toc203414565)

# **1. METHODS**

## **1.1 Protocol**

The review protocol has been registered on PROSPERO and is available from: https://www.crd.york.ac.uk/prospero/display_record.php?ID=CRD42022356862

**Changes to the protocol:**

1. For risk of bias, a modified version of the NIH Quality Assessment Tool for Case Series Studies was used. “Adequate length of follow up” was removed as it was deemed not applicable.
2. Image resolution changed from 1.5 x 1.5 x 1.5 mm to 1 x 1 x 1 mm
3. Functionnectome mapping performed
4. Additional visualization tools used, including: Inkscape, MRIcroGL and FSL’s HCP1065 standard-space FA atlas.

**Review question**

For patients undergoing neurosurgery, what is the relationship between positive direct electrical stimulation (DES) sites in a standard space and observed or reported behaviour?

**Searches**

Sources: PubMed, Embase (via Ovid), Web of Science.

Studies are included if the primary study design falls into one of the follow categories: case studies, clinical trials, cohort studies, case reports, and/or case-control.

Additional search strategy information can be found in the attached PDF document (link provided below).

**Search strategy**

https://www.crd.york.ac.uk/PROSPEROFILES/356862_STRATEGY_20220902.pdf

**Types of study to be included**

Studies are included if the primary study design falls into one of the follow categories: case studies, clinical trials, cohort studies, case reports, and/or case-control.

**Condition or domain being studied**

Collating and synthesising positive direct electrical stimulation (DES) sites to generate a novel brain map has yet to be performed. The objective of the present paper is to generate a novel functional anatomical map based on positive DES stimulation sites via a systematic review of the literature.

**Participants/population**

The participants/population includes patients undergoing neurosurgery.

**Intervention(s), exposure(s)**

The exposure includes use of direct electrical stimulation (DES) with reports of unambiguous MNI or Talairach coordinates.

**Comparator(s)/control**

Not applicable.

**Main outcome(s)**

The analysis aims to establish a spatial relationship between a location in standard space (e.g., MNI) and an observed or reported behaviour. In this meta-analytic study, published DES loci and their associated behavioural outcome will be gathered, grouped into definitive behaviours and modelled separately to generate focal and disconnectomic maps of disrupted behaviour.

**Measures of effect**

Standard procedures embedded in SPM will be applied, reporting peak activation loci and their corresponding t-statistic and FWE p-values.

**Additional outcome(s)**

None.

**Measures of effect**

Not applicable.

**Data extraction (selection and coding)**

Studies are included if the primary study design falls into one of the follow categories: case studies, clinical trials, cohort studies, case reports, and/or case-control. Eligible studies require (a) use of direct electrical stimulation (DES) on patients and (b) reports of clear numerical MNI or Talairach coordinates. The search was limited to studies in peer-reviewed journals, published in English. No exclusion criteria were set for publication dates.

A positive/responsive site is defined by a point that either disrupted, annulled, or increased an already present function via DES. Positive DES sites were reported in one system of coordinates to facilitate comparisons between studies. This was achieved by translating findings from studies reporting coordinates in the Talairach system into MNI coordinates via the Bioimage Talairach to MNI converter, an application built through components of the Yale BioImage Suite Package. The coordinate system used most commonly across the included DES points will be the applied across all points, in order to minimise potential error from the translation process.

Grouping DES points based on behavioural associations will consider (i) the original study author(s) assigned grouping, (ii) consensus between authors independently coding data (AA, VS), and (iii) the ability of different intraoperative tasks to elicit the same behaviour based on the scientific literature.

**Risk of bias (quality) assessment**

A risk of bias assessment will be carried out using the NIH Quality Assessment Tool for Case Series Studies.

**Strategy for data synthesis**

Meta-analytic loci will be gathered and grouped by their reported behavioural associations. For each location, a corresponding image (1.5×1.5×1.5 mm isotropic sampling) will then be generated with an intensity of zero at all locations except the stimulated location where intensity is one. Each image will be convolved with a 3D Gaussian kernel of pre-determined width to enable modelling of spatial uncertainty in the location of the stimulation and approximate the local distribution of focally induced disruption. This approach facilitates group analysis of sparse data by accounting for between-subject variation in functional-anatomical relationships not captured by anatomical registrations, analogous to the approach used in meta-analytic modelling of functional activation data (Eickhoff et al. 2009; Eickhoff et al. 2012).

In the more anatomically expressive, connective form, the transformation is achieved by a probabilistic projection of the distributed connectivity of each locus, incorporating not just the uncertainty but also the network distribution of the disruption. To achieve this, large-scale high-resolution 7T diffusion data (n=178) obtained from the Human Connectome Project, for which probabilistic tractography was applied to derive local fibre orientation information, will be used as priors. Each stimulation point, now treated as a lesion, will be a seed point from which WM tracts are projected, resulting in a probabilistic map of the distributed networks supporting a behaviour elicited in the seed point (Foulon et al. 2018).

For each behavioural condition of interest separately, stimulation and disconnectomic maps will be entered into a voxel-wise general linear model with electrode density as the dependent variable and the binary behavioural effect as the independent variables. A planned one-tailed voxel-wise t-test of each behavioural condition will be performed and thresholded at p<0.05 FWE (peak voxel) to account for multiple comparisons.

Image processing and spatial inference will be performed using SPM12 (http://www.fil.ion.ucl.ac.uk/spm/) and visualisation using SurfIce toolbox (https://www.nitrc.org/projects/surfice).

**Analysis of subgroups or subsets**

None planned.

**Contact details for further information**

Viktoria Sefcikova

viktoria.sefcikova.18@ucl.ac.uk

Organisational affiliation of the review

University College London

**Review team members and their organisational affiliations**

Dr Anna Alexandratou. Neurology Department, Evangelismos Hospital, Athens, Greece; UCL Queen Square Institute of Neurology, University College London, Queen Square, London, WC1N 3BG, United Kingdom

Dr Viktoria Sefcikova. UCL Queen Square Institute of Neurology, University College London, Queen Square, London, WC1N 3BG, United Kingdom; The University of Queensland Medical School, Brisbane, QLD, Australia; Caboolture Hospital, Caboolture, Australia

Michael Elmalem. UCL Department of Brain Repair and Rehabilitation High Dimensional Neurology Group Queen Square Institute of Neurology Queen Square, London, United Kingdom; Max Planck Institute for Human Cognitive and Brain Sciences Leipzig, Germany

Dr Jeroen Bisschop. Institute of Diagnostic and Interventional Radiology, University Hospital Zurich, University of Zurich, Zurich, Switzerland

Dr Sibel Huet. UCL Queen Square Institute of Neurology, University College London, Queen Square, London, WC1N 3BG, United Kingdom

Dr George Samandouras. UCL Queen Square Institute of Neurology, University College London, Queen Square, London, WC1N 3BG, United Kingdom; Victor Horsley Department of Neurosurgery, The National Hospital for Neurology and Neurosurgery, Queen Square, London, United Kingdom

Professor Parashkev Nachev. High Dimensional Neurology Group, UCL Queen Square Institute of Neurology, University College London, Russell Square House, Bloomsbury, London, United Kingdom

**Type and method of review**

Meta-analysis, Systematic review

**Anticipated or actual start date**

03 September 2022

**Anticipated completion date**

31 October 2022

**Funding sources/sponsors**

Michael Elmalem is funded by the Max Planck Institute for Human Cognitive and Brain Sciences and the UCLH NIHR Biomedical Research Centre. Parashkev Nachev is funded by Wellcome and the UCLH NIHR Biomedical Research Centre.

**Conflicts of interest**

None known

**Language**

English

**Country**

Australia, England, Germany

**Stage of review**

Review Ongoing

**Subject index terms status**

Subject indexing assigned by CRD

**Subject index terms**

Brain Mapping; Cognition; Humans; Motor Cortex

**Date of registration in PROSPERO**

06 September 2022

**Date of first submission**

04 September 2022

## **1.2 Search strategy**

**PubMed:**

“Direct electrical stimulation” OR "direct electrostimulation" OR “direct electric stimulation”

OR “electric brain stimulation” OR “direct cortical stimulation” OR “electrical brain

stimulation” OR “electrical stimulation mapping” OR “electrocortical stimulation” OR “cortical

stimulation”

**Embase (via Ovid):**

(1) direct electric* stimulation.mp.

(2) electric* brain stimulation.mp.

(3) direct cortical stimulation.mp.

(4) electrical stimulation mapping.mp.

(5) cortical stimulation mapping.mp.

(6) electrocortical stimulation.mp.

(7) cortical stimulation.mp.

(8) direct electrostimulation.mp.

(9) or/1-8

[mp=title, abstract, heading word, drug trade name, original title, device manufacturer, drug

manufacturer, device trade name, keyword, floating subheading word, candidate term word]

**Web of Science:**

TS=("direct electric* stimulation" OR "electric* brain stimulation" OR "direct cortical

stimulation" OR "electrical stimulation mapping" OR "cortical stimulation mapping" OR

"electrocortical stimulation" OR "direct electrostimulation")

# **2. RESULTS**

## **2.1 PRISMA flow diagram depicting the study selection process**

The study selection process was conducted according to the Preferred Reporting Items for Systematic Reviews and Meta-Analyses (PRISMA) guidelines (Page et al. 2021). The ‘Reports not retrieved’ fell into one or more of the following categories: articles not in the English language, abstracts-only or no full text available, lack of publication in a peer review journal, or full text was unable to be located.

Records screened

(n=6015)

Records identified from:

PubMed (n=4282)

Embase via Ovid (n=5343)

Web of Science (n=2346)

Records removed *before screening*:

Duplicate records removed using EndNote X9 (n=5912)

Duplicate records manually removed (n=44)

Records excluded

(n=5129)

Reports sought for retrieval

(n=886)

Reports not retrieved

(n=82)

Reports assessed for eligibility

(n=804)

Reports excluded:

- Stimulation method not eligible (n=367)

- Book chapter (n=2)

- Video publication (n=2)

- Significant overlap in stimulation coordinates (n=4)

- Lack of clear numerical MNI or Talairach coordinates for positive sites (n=408)

Studies included in review

(n=21)

**Identification of studies via databases and registers**

**Identification**

**Screening**

**Included**

## **2.2 Risk of bias table of included studies**

*A “consecutive series” refers to a study that includes all eligible participants identified by the researchers in a pre-defined time period. For the risk of bias assessment, a study qualified as a ‘consecutive series’ when the term was explicitly mentioned in the manuscript, or the described recruitment of participants reflected the premise of a consecutive series.

|  | Clear objective | Population clearly described | Consecutive series* | Intervention clearly described | Clearly measured outcomes and reliable | Statistical methods well described | Results well described |
| --- | --- | --- | --- | --- | --- | --- | --- |
| Mani et al. (2008) | + | - | - | + | + | - | + |
| Roux et al. (2009) | + | + | - | + | + | + | + |
| Matsumoto et al. (2011) | + | + | - | + | + | - | + |
| Roux et al. (2012) | + | + | - | + | + | + | + |
| Roux et al. (2014) | + | + | - | + | + | + | + |
| Tate et al. (2014) | + | + | + | + | + | + | + |
| Wu et al. (2015) | + | + | - | + | + | + | + |
| Roux et al. (2015) | + | + | - | + | + | + | + |
| Rech et al. (2016) | + | + | - | + | + | - | + |
| Herbet et al. (2017a) | + | + | - | + | + | + | + |
| Herbet et al. (2017b) | + | + | - | + | + | + | + |
| Chang et al. (2017) | + | + | + | + | + | + | + |
| Zaca et al. (2018) | + | + | - | + | + | + | + |
| Yordanova et al. (2019) | + | + | - | + | + | + | + |
| Mandonnet et al. (2019) | + | + | + | + | + | + | + |
| Sarubbo et al. (2020) | + | + | - | + | + | + | + |
| Schrouff et al. (2020) | + | + | - | + | + | + | + |
| Simone et al. (2021) | + | + | - | + | + | + | + |
| Zhou et al. (2021) | + | + | + | + | + | + | + |
| Ng et al. (2021) | + | + | + | + | + | + | + |
| Giampiccolo et al. (2022) | + | + | + | + | + | + | + |

**2.3 Characteristics of included studies**

†Exact ages were not provided; therefore, standard deviation could not be calculated. Range is provided in brackets instead and an approximate mean was calculated using the given extreme values. ¶ Calculated based on data available for seven out of eight subjects. NA=not available; F=female; M=male; R=right; L=left; Amb=ambidextrous; SD=standard deviation; LGG=low grade glioma; HGG=high grade glioma; CA=cavernous angiomas; SFG=superior frontal gyrus; MFG=middle frontal gyrus; IFG=inferior frontal gyrus; VON=visual object naming; VWFA=visual word form area

| **First author, year** | **N** | **Age**  **(mean ± SD)** | **Sex** | | **Handedness** | | | **Stimulation method** | **Lesion pathology** | **Hemisphere** | | **Details of anatomical sites** |
| --- | --- | --- | --- | --- | --- | --- | --- | --- | --- | --- | --- | --- |
|  |  |  | F | M | R | L | A |  |  | R | L |  |
| Mani, 2008 | 3 | NA | NA | NA | 2 | 1 | 0 | Subdural | Epileptogenic foci | 2 | 1 | Dominant hemisphere |
| Roux, 2009 | 12 | 37.1 ± 8.4 | 5 | 7 | 10 | 2 | 0 | DES | 1 LGG, 11 NA | 1 | 11 | Frontal |
| Matsumoto, 2011 | 2 | 31 ± 1 | 2 | 0 | 1 | NA | NA | Subdural | Epileptogenic foci | 0 | 2 | Temporal |
| Roux, 2012 | 14 | 42 ± 18.6 | 3 | 11 | 14 | 0 | 0 | DES | 5 LGG, 6 HGG, 2 CA, 1 NA | 0 | 14 | Posterior Sylvian fissure |
| Roux, 2014 | 30 | 47.4 ± 15.9 | 9 | 21 | 27 | 3 | 0 | DES | 9 LGG, 15 HGG, 6 NA | 7 | 23 | Temporo-parietal |
| Tate, 2014 | 165 | 38.7 ± 10.4 | 74 | 91 | 139 | 22 | 4 | DES | 165 LGG | NA | NA | Majority frontal (n=43) and temporal (n=25) |
| Wu, 2015 | 66 | 41.8 ± 12.7 | 23 | 43 | 66 | 0 | 0 | DES | 44 LGG, 22 HGG | 0 | 66 | Peri-insular |
| Roux, 2015 | 90 | 48 ± 10.4 | 47 | 43 | 87 | 3 | 0 | DES | NA | 13 | 77 | NA |
| Rech, 2016 | 18 | 31.9 ± 9.2 | 9 | 9 | 15 | 2 | 1 | DES | 18 LGG | 10 | 8 | NA |
| Herbet, 2017a | 13 | 44.7 ± 12.8 | 5 | 8 | 12 | 1 | 0 | DES | 13 LGG | 13 | 0 | Temporal (n=8), frontal (n=5), parietal (n=4), insular (n=4) |
| Herbet, 2017b | 3 | 47.3 ± 7.0 | 3 | 0 | 3 | 0 | 0 | DES | 3 LGG | 3 | 0 | Frontal (n=3), temporal (n=1), insular (n=1) |
| Chang, 2017 | 102 | 41 (14-84)† | 42 | 60 | NA | NA | NA | DES | 102 LGG | 98 | 4 | Temporal (n=29), insula (n=29), frontal (n=20), parietal (n=11), multiple lobes (n=13) |
| Zacà, 2018 | 7 | 41.4 ± 5.4 | 3 | 4 | 7 | 0 | 0 | DES | 4 LGG, 3 CA | 4 | 3 | Frontal (n=6), temporal (n=1) |
| Yordanova, 2019 | 23 | 38 ± 12.8 | 8 | 15 | 18 | 3 | 2 | DES | 23 LGG | 23 | 0 | Frontal (n=20), temporal (n=6), insular (n=6), occipital (n=1) |
| Mandonnet, 2019 | 21 | 34.4 ± 7 | 5 | 16 | 20 | 1 | 0 | DES | 17 LGG, 4 HGG | 0 | 21 | Frontal (n=14), temporal (n=10), insular (n=5), parietal (n=1) |
| Sarubbo, 2020 | 256 | 38.7 ± 10.3 | 121 | 135 | 218 | 24 | 14 | DES | 256 LGG | 101 | 155 | Varied, majority frontal (n=177) and temporal (n=143) |
| Schrouff, 2020 | 8 | 44.2 ± 15.4¶ | 2 | 6 | 6 | 1 | NA | Subdural | Epileptogenic foci | 5 | 2 | NA |
| Simone, 2021 | 46 | 16-65† | 24 | 22 | 44 | 2 | 0 | DES | 46 LGG | 26 | 20 | Near the hand-knob area |
| Zhou, 2021 | 33 | 39 ± 10 | 13 | 20 | 33 | 0 | 0 | DES | 27 LGG, 6 HGG | 0 | 33 | Frontal (n=17), insular (n=8), Temporal (n=5), parietal (n=2), fronto-temporal (n=1) |
| Ng, 2021 | 25 | 39.4 ± 11.4 | 6 | 19 | 25 | 0 | 0 | DES | 25 LGG | 0 | 25 | Near the VWFA |
| Giampiccolo, 2022 | 17 | 38.7 ± 14.5 | 9 | 8 | 14 | 3 | 0 | DES | 17 LGG | 0 | 17 | Insula (n=10), temporal (n=9), frontal (n=4), parietal (n=2) |

## **2.4 Intraoperative tasks**

FEF=frontal eye field; DES=direct electrical stimulation.

| **Task name** | **Task definition** | **Behavioral finding associated with a “positive stimulation site”** | **Included studies** |
| --- | --- | --- | --- |
| Stimulation motor mapping | While the patient is at rest, DES is applied to brain areas responsible for muscle movement | Overt muscle twitch which occurs during stimulation | Roux et al. 2009; Sarubbo et al. 2020; Simone et al. 2021; Tate et al. 2014 |
| Stimulation eye-movement mapping | While the patient is at rest, DES is applied to the cortical FEF or the white matter beneath | Altered eye movements (e.g., anterior FEF stimulation leading to horizonal eye movement, posterior FEF leading to upward movement, and white matter leading to conjugate contraversive ocular deviation) | Sarubbo et al. 2020 |
| Continuous complex motor task | Patient performs a continuous, complex motor task (e.g., simultaneous flexion-extension of the hand, arm, and forearm) using the limb contralateral to the stimulation site | Altered task performance, such as acceleration, deceleration, or halting movement | Rech et al. 2016; Sarubbo et al. 2020; Zhou et al. 2021 |
| Stimulation sensory mapping | While the patient is at rest, DES is applied to brain areas responsible for sensation | Verbally reported sensory changes (e.g., dysesthesias of the contralateral face, arm, or leg) | Chang et al. 2017; Sarubbo et al. 2020; Tate et al. 2014 |
| Visual object naming | Patient names aloud drawings of various objects | Global behavioral arrest where the patient stops speaking during stimulation all together | Roux et al. 2015 |
| Visual object naming | Patient names aloud drawings of various objects | Anomia was defined as: (A) the patient says they know the object but cannot find the target word (the stopping of stimulation allows the target word to be produced) (Roux et al. 2015) or (B) “pure anomia” which is not related to motor/praxis/visual disturbances (Chang et al. 2017; Ng et al. 2021; Sarubbo et al. 2020) | Chang et al. 2017; Ng et al. 2021; Sarubbo et al. 2020; Roux et al. 2015 |
| Visual object naming | Patient names aloud drawings of various objects | Speech arrest or “anarthria” which was defined as: (A) complete cessation of speech output with retained ability to perform alternative and regular tongue movements (Sarubbo et al. 2020; Tate et al. 2014; Zacà et al. 2018) or (B) if patients are unable to say a leading sentence to picture naming “This is a…” (Sarubbo et al. 2020; Tate et al. 2014) | Sarubbo et al. 2020; Tate et al. 2014; Zacà et al. 2018 |
| Visual object naming | Patient names aloud drawings of various objects | Dysarthria or improper articulation with retention of verbal output (Tate et al. 2014) | Tate et al. 2014 |
| Visual object naming | Patient names aloud drawings of various objects | Perseverations which was defined as initially correctly stating the object presented (e.g., “This is a hammer” but when presented with a different object after a delay, the patient incorrectly states the previous response (i.e., “This is a hammer.” (Sarubbo et al. 2020) Alternatively, the preservation can be related to a recent word, phoneme, and rarely a more distant item (Mandonnet et al. 2019) | Mandonnet et al. 2019; Sarubbo et al. 2020 |
| Visual object naming | Patient names aloud drawings of various objects | Phonological paraphasia or interference which was defined in the following ways: (A) target word identified but incorrectly pronounced (Roux et al. 2015); (B) target incorrectly identified with phonemes substituted or rearranged (Ng et al. 2021; Sarubbo et al. 2020); (C) production of a phonologically disturbed word (Herbet et al. 2016); or (D) production of a non-word (Giampiccolo et al. 2022) | Roux et al. 2015; Ng et al. 2021; Sarubbo et al. 2020; Herbet et al. 2016; Giampiccolo et al. 2022 |
| Visual object naming | Patient names aloud drawings of various objects | Semantic paraphasia or interference (i.e., producing an incorrect word which is semantically-related, e.g., saying “dog” when presented with a picture of a cat) | Herbet et al. 2017a; Ng et al. 2021; Sarubbo et al. 2020 |
| Visual object naming | Patient names aloud drawings of various objects | Hesitation, meaning the patient did not produce the correct response, hesitated, and then were uncertain of their final response | Roux et al. 2015 |
| Counting or recitation test | Patient either counts from 0 to 10 or recites the days of the week | Speech arrest (i.e., speech has stopped, but the patient retains the ability to perform alternative and regular tongue movements) | Chang et al. 2017; Mandonnet et al. 2019; Rech et al. 2016; Sarubbo et al. 2020; Tate et al. 2014 |
| Reading | Patient instructed to either (A) read aloud unrelated and unrehearsed sentences; (B) read various regular and irregular words; (C) read a story; or (D) read numbers | Reading impairment and/or alexia | Mani et al. 2008; Roux et al. 2009; Roux et al. 2012; Roux et al. 2014 |
| Pseudoword reading | Patient presented pseudowords and instructed to read | Reading deficit for pseudowords | Roux et al. 2012; Roux et al. 2014 |
| ECLA 16+ test | A battery of regular, irregular, and pseudowords are presented to the patient consecutively (every 4 seconds) on a screen for reading | Three deficit patterns noted: (1) pure alexia (i.e., impaired reading of all three word types without additional spoken language disturbances), (2) phonological alexia (i.e., DES-related impairment of pseudoword reading specifically), and (3) lexical-semantic alexia (i.e., only irregular word reading is impaired upon DES) | Ng et al. 2021 |
| Writing | Handwriting tasks included either (A) writing simple sentences to dictation (Roux et al. 2009; Roux et al. 2014); or (B) copying (isolated letters, words, and numbers) (Roux et al. 2009) | Pure agraphia was defined either by (1) impaired grapheme production, slow writing, or arrest of writing (Roux et al. 2009); or (2) writing disturbance (phonologic or semantic) with no deficits in understanding, naming or reading (Roux et al. 2014) | Roux et al. 2009; Roux et al. 2014 |
| The Pyramids and Palm Trees Test | The patient is shown an image, and asked to finger point to the semantically linked image out of two options | Incorrect image matching | Sarubbo et al. 2020; Herbet et al. 2017a |
| Visual two-picture match | The patient is shown a central image, and asked to associate the image with one of the four images depicted peripherally via finger-pointing | Incorrect image matching | Roux et al. 2015 |
| Confrontational naming | Patient is shown four different images (e.g., flower, boat, dog, hammer) and asked “Show me where is the boat?” | Incorrect word to image matching | Roux et al. 2014 |
| Auditory word-picture matching | Four objects are visually presented to the patient. The patient is given in auditorily perceived word with the carrier sentence “Where is the…” and instructed to match the word to the picture. Stimulation is applied 2 seconds prior to the question. | Two types of interference patterns identified: (A) patient did not have sudden hearing loss, but complained about intelligibility of the sentence; and (B) patient unaware of errors on identifying the image corresponding to the word. The DES site was specific to auditory comprehension when there was no interference in vision or object naming at the same site. | Roux et al. 2015; Mani et al. 2008 |
| Auditory object naming | The patient listens to a sound, then names the sound (e.g., “doorbell ringing”) | Inability to identify the auditorily presented sound | Roux et al. 2015 |
| Repetition | Patient is instructed to repeat a dictated sentence | Inability to repeat the sentence | Roux et al. 2014; Sarubbo et al. 2020 |
| Auditory comprehension | Patient was auditorily given a one-sentence command (e.g., “open your mouth”) | Failure to perform gestures | Matsumoto et al. 2011; Roux et al. 2014 |
| Token test | Two-step verbal directions are provided to the patient regarding selecting drawings which vary based on color, shape, and size. For example, the two-steps for a DES stimulation could include selection of the drawing that is “small blue square” and “large red round”. | Impairment involved either (A) speech discrimination interference (i.e., inability to understand the auditorily presented content), or (B) inaccurate task performance with intact perception of the auditory instruction | Ng et al. 2021; Roux et al. 2015 |
| Reading the Mind in the Eyes | The patient is shown a photo of the eye region and asked to verbally state the corresponding mental state from a list of four adjectives | Mentalizing impairment, which was defined as either (A) incorrect mental state selected or (B) absence of a response (i.e., response time >12 seconds) (Herbet et al. 2015) | Sarubbo et al. 2020 |
| Adapted Reading the Mind in the Eyes | The patient is shown a photo of the eye region and verbally states the mental state description from two adjective options | Mentalizing impairment, which was defined as either (A) incorrect mental state selected or (B) answer provided after 5 seconds | Yordanova et al. 2019 |
| Line bisection | Patient is shown a black line, aligned to their eye axis, and asked to mark the midpoint of the line with a pen | Deviation from the midpoint of the line | Tate et al. 2014; Herbet et al. 2017b; Sarubbo et al. 2020 |
| Stimulation visual mapping | While the patient is at rest, DES is applied to the primary visual cortex or optic radiations | Visual disturbance (e.g., blurring, “phosphenes”, impression of a “shadow”) | Sarubbo et al. 2020 |
| Opposite quadrant visual object naming | Patient names aloud pictures which have been placed diagonally, in opposite quadrants of the visual field, while DES is applied to the visual cortex | Transient visual disturbances within the contralateral visual hemifield (blurred vision, phosphenes, shadow) preventing subjects from naming the picture situated in the contralateral quadrant | Sarubbo et al. 2020 |
| Face and non-face perception | Patient is presented with images of faces (human, mammal, bird, and marine), and non-faces, including bodies without faces (same four categories), limbs (human), objects, and places | Changes in face perception i.e. distorted face, change of shape of face, change of eyes | Schrouff et al. 2020 |

## **2.4 Behaviours**

The components of each of the seven behavioural categories in a stimulation context are defined in further detail.

| **Behavioural category** | **Subcategory** | **Definition** |
| --- | --- | --- |
| Motor | Movement | ‘Movement’ involves muscle contractions. Intraoperative, a ‘positive’ site is characterised by involuntary muscle actions induced by stimulation (e.g., eye movements, overt muscle twitch or contraction). |
| Motor | Motor control | ‘Motor control’ includes control of voluntary movements, such as initiation or continuation of voluntary movements. A ‘positive’ site is identified when electrostimulation applied during a motor task leads to alterations in task performance (e.g., acceleration, deceleration, or halting of movement). |
| Somatosensory | N/A | Somatosensation is the perception of external or internal stimuli, and the regulation of body position and balance (proprioception). ‘Positive’ sites involved verbally reported sensory changes (e.g., dysesthesias). |
| Speech and language | Articulation | Included ‘speech arrest’ / ‘anarthria’ and ‘dysarthria’, ‘verbal apraxia’ and ‘perseveration’. Speech arrest/anarthria is the complete cessation of speech output with retained ability to perform alternative and regular tongue movements. Dysarthria is improper articulation with retention of verbal output. Verbal apraxia is impaired sequencing of movements required for speech production (without muscle weakness or language comprehension deficits). Perseveration is the repetition of a previous verbal response when no longer relevant. |
| Speech and language | Phonology | ‘Phonology’ refers to the correct organisation and articulation of speech sounds (phonemes) during spoken word production. It involves correct pronunciation of a word in response to a stimulus, including correct ordering and combination of phonemes to form intelligible speech. |
| Speech and language | Reading | ‘Reading’ refers to the ability to accurately decode and comprehend written language, including words, sentences, and numbers. It involves both regular and irregular word reading, with the latter requiring access to stored lexical knowledge rather than phonetic decoding alone. |
| Speech and language | Naming | ‘Naming’ is the ability to retrieve and produce the correct word corresponding to a specific object. It reflects intact lexical-semantic processing and is independently of impairments in motor function, praxis, or visual perception. |
| Speech and language | Neologistic | ‘Neologistic’ refers to the production of non-words that are often phonologically related to real words, but are not recognisable as actual words in the speaker’s language. |
| Speech and language | Verbal semantics | ‘Verbal semantics’ refers to the processing and understanding of meaning through language, specifically via spoken or written words. |
| Non-verbal semantics | N/A | ‘Non-verbal semantics’ refers to the ability to understanding and processing meaning through non-linguistic representations (e.g., pictures). It does not involve spoken or written language—hence focusing on conceptual knowledge and associative meaning via visual stimuli. |
| Auditory comprehension | N/A | ‘Auditory comprehension’ involves the ability to process and interpret the meaning of spoken output (e.g, correct picture selection in response to a heard sentence, or performing the correct gesture in response to a heard command). |
| Mentalizing | N/A | ‘Mentalizing’ involves the ability to understand human behaviour in terms of complex affective (e.g., mental state associated with the eye region of a human face). |
| Visuospatial | Spatial perception | ‘Spatial perception’ is defined as the ability to understand the relationship, including position, between objects in space. This includes correct identification of an item’s midpoint and its limits in a given space. |
| Visuospatial | Vision | ‘Vision’ involves the ability to perceive the world through the eyes in each of the four quadrants. |
| Visuospatial | Face perception | ‘Face perception’ involves the ability recognise and interpret faces, including distinguishing between human vs. non-human faces. |
| Visuospatial | Writing | ‘Writing’ involves the ability to form meaningful letters or characters relevant to language. This may include correct handwriting of simple sentences to dictation, or copying isolated letters, worse, or numbers. |

## **2.5 Stimulations sites and Barycentre coordinates associated with each function**

L=left; R=right; NA=not available; POp=pars opercularis. The anatomical site of the cortical barycentric locations were based on the Harvard-Oxford Brain Atlas and John Hopkins University (JHU) (Mori et al. 2005). The ‘central opercular cortex’ is a region emerged from the atlases employed and refers to a broader territory containing the frontal operculum. We have, therefore, opted to refer to the region as “central opercular cortex (POp)”.

| **Function (hemisphere)** | **Stimulation sites** | **Barycentre MNI coordinate**  **x y z** | | | **Anatomical location** |
| --- | --- | --- | --- | --- | --- |
| Movement (L) | 404 | -47 | -7 | 43 | Precentral gyrus |
| Movement (R) | 412 | 45 | -6 | 45 | Precentral gyrus |
| Motor control (L) | 143 | -43 | 5 | 39 | Middle frontal gyrus |
| Motor control (R) | 131 | 36 | 5 | 34 | Precentral gyrus |
| Somatosensory (L) | 134 | -50 | -19 | 48 | Postcentral gyrus |
| Somatosensory (R) | 222 | 47 | -21 | 46 | Postcentral gyrus |
| Speech arrest, anarthria, dysarthria (L) | 963 | -61 | 4 | 22 | Precentral gyrus |
| Speech arrest, anarthria, dysarthria (R) | 165 | 61 | 2 | 24 | Precentral gyrus |
| Verbal apraxia (L) | 116 | -56 | -2 | 27 | Precentral gyrus |
| Verbal apraxia (R) | 68 | 58 | 4 | 28 | Precentral gyrus |
| Perseveration (L) | 46 | -25 | 16 | 9 | Anterior limb of internal capsule, Putamen |
| Perseveration (R) | 1 | 23 | 22 | 4 | Anterior limb of internal capsule |
| Phonologic (L) | 147 | -46 | -12 | 8 | Central opercular cortex (POp) |
| Phonologic (R) | 9 | 43 | -12 | 1 | Insular cortex |
| Reading (L) | 150 | -59 | -33 | 9 | Posterior superior temporal gyrus |
| Reading (R) | 2 | 49 | -52 | -12 | Posterior middle temporal gyrus |
| Naming (L) | 564 | -61 | -12 | 11 | Central opercular cortex (POp) |
| Naming (R) | 18 | 55 | -21 | 16 | Parietal opercular cortex |
| Neologistic (L) | 12 | -45 | -24 | 3 | Heschl’s gyrus |
| Neologistic (R) | 0 | NA | | | NA |
| Verbal Semantics (L) | 162 | -45 | -8 | 3 | Insular cortex |
| Verbal Semantics (R) | 8 | 38 | -2 | -1 | Insular cortex |
| Non-Verbal Semantics (L) | 46 | -55 | -15 | 9 | Central opercular cortex (POp) |
| Non-Verbal Semantics (R) | 44 | 43 | -8 | 12 | Central opercular cortex (POp) |
| Auditory comprehension (L) | 114 | -60 | -18 | 7 | Posterior superior temporal gyrus |
| Auditory comprehension (R) | 1 | 53 | -23 | -3 | Posterior superior temporal gyrus |
| Mentalizing (L) | 0 | NA | | | NA |
| Mentalizing (R) | 41 | 47 | 20 | 31 | Middle frontal gyrus |
| Spatial Perception (L) | 7 | -56 | -35 | 37 | Supramarginal gyrus |
| Spatial Perception (R) | 51 | 58 | -29 | 21 | Parietal opercular cortex |
| Vision (L) | 18 | -32 | -64 | 1 | Optic radiation |
| Vision (R) | 25 | 37 | -47 | 2 | Optic radiation |
| Writing (L) | 73 | -64 | -29 | 18 | Anterior division of supramarginal gyrus |
| Writing (R) | 1 | 12 | -4 | 76 | Superior frontal gyrus |
| Face perception (L) | 2 | -28 | -55 | -22 | Fusiform gyrus |
| Face perception (R) | 25 | 38 | -57 | -23 | Fusiform gyrus |

## **2.6 Statistical values and anatomical location of clusters of different functions**

Not generated means not enough stimulation points were available for a map to be created. No suprathreshold clusters means no values overcame the p-value<0.05 threshold. L=left; R=right

| **Function (hemisphere)** | **t-statistic** | **p value  (FWE corrected)** | **MNI Coordinates**  **x y z** | | | **Anatomical Location** |
| --- | --- | --- | --- | --- | --- | --- |
| Movement (L) | 8.82 | 0.000 | -58 | 0 | 34 | Precentral gyrus |
|  | 8.44 | 0.000 | -39 | -16 | 68 | Precentral gyrus |
|  | 6.13 | 0.000 | -51 | -8 | 57 | Precentral gyrus |
| Movement (R) | 7.95 | 0.000 | 39 | -15 | 66 | Precentral gyrus |
|  | 7.29 | 0.000 | 60 | 3 | 34 | Precentral gyrus |
|  | 7.02 | 0.000 | 57 | -2 | 42 | Precentral gyrus |
| Motor control (L) | 6.67 | 0.000 | -62 | 4 | 32 | Precentral gyrus |
| Motor control (R) | 4.17 | 0.045 | 14 | 6 | 36 | Cingulate gyrus, anterior division |
| Somatosensory (L) | 5.59 | 0.000 | -50 | -20 | 54 | Postcentral gyrus |
|  | 5.14 | 0.001 | -50 | -24 | 62 | Postcentral gyrus |
|  | 5.07 | 0.001 | -58 | -9 | 34 | Postcentral gyrus |
| Somatosensory (R) | 6.30 | 0.000 | 64 | -10 | 33 | Postcentral gyrus |
|  | 5.96 | 0.000 | 58 | -14 | 45 | Postcentral gyrus |
|  | 5.64 | 0.000 | 46 | -26 | 60 | Postcentral gyrus |
| Speech arrest (L) | 21.10 | 0.000 | -64 | 4 | 15 | Precentral gyrus |
| Speech arrest (R) | 7.93 | 0.000 | 69 | 2 | 16 | Precentral gyrus |
|  | 4.92 | 0.003 | 60 | 3 | 34 | Precentral gyrus |
|  | 4.20 | 0.035 | 51 | -6 | 44 | Precentral gyrus |
| Naming (L) | 8.69 | 0.000 | -69 | -26 | 6 | Superior temporal gyrus, posterior division |
|  | 8.38 | 0.000 | -69 | -32 | 12 | Superior temporal gyrus, posterior division |
|  | 7.65 | 0.000 | -64 | -14 | -3 | Superior temporal gyrus, posterior division |
| Naming (R) | No suprathreshold clusters | | | | |  |
| Neologistic (L) | No suprathreshold clusters | | | | |  |
| Auditory comprehension (L) | 5.62 | 0.000 | -58 | -22 | 4 | Superior temporal gyrus, posterior division |
|  | 5.12 | 0.001 | -57 | -2 | -3 | Superior temporal gyrus, anterior division |
|  | 4.97 | 0.002 | -60 | 0 | -12 | Superior temporal gyrus, anterior division |
| Auditory comprehension (R) | Not generated |  |  |  |  |  |
| Perseveration (L) | 5.30 | 0.002 | -22 | 27 | 9 | Frontal white matter |
|  | 4.99 | 0.004 | -27 | 15 | 4 | Putamen |
|  | 4.53 | 0.018 | -18 | 21 | 16 | White matter close to the head of caudate |
| Perseveration (R) | Not generated | | | | |  |
| Mentalizing (L) | Not generated | | | | |  |
| Mentalizing (R) | No suprathreshold clusters | | | | |  |
| Face perception (L) | Not generated | | | | |  |
| Face perception (R) | 7.25 | 0.000 | 39 | -57 | -16 | Posterior fusiform gyrus |

## **2.7 Statistical values and anatomical location of disconnectome clusters of different behaviours**

L=left; R=right

| **Behaviour** | **t-stat** | **p value  (FWE corrected)** | **MNI Coordinate x y z** | | | **Anatomical Location** |
| --- | --- | --- | --- | --- | --- | --- |
| **Movement (L)** | 21.58 | 0.000 | -19 | -10 | 28 | No label found |
|  | 5.02 | 0.005 | 10 | 16 | 63 | 20% Frontal Aslant Tract R, 15% Superior Longitudinal Fasciculus 1 R |
|  | 4.74 | 0.043 | -28 | 19 | -6 | No label found |
| **Movement (R)** | 22.23 | 0.000 | 20 | -13 | 27 | 7% Corticospinal Tract R, 6% Superior Thalamic Radiation R |
|  | 4.78 | 0.010 | 54 | 20 | -1 | No label found |
|  | 4.37 | 0.046 | 19 | 23 | 40 | 7% Frontal Aslant Tract R |
| **Motor Control (L)** | 9.72 | 0.000 | -30 | -7 | 29 | 54% Superior Thalamic Radiation L, 6% Corticospinal Tract L |
|  | 4.89 | 0.017 | -20 | -16 | 52 | 40% Superior Longitudinal Fasciculus 1 L, 26% Superior Thalamic Radiation L, 10% Corticospinal Tract |
|  | 4.74 | 0.029 | 28 | -11 | 23 | 58% Corticospinal Tract R, 38% Superior Thalamic Radiation R |
|  | 4.62 | 0.044 | 37 | 20 | 36 | 7% Superior Longitudinal Fasciculus II R |
| **Motor Control (R)** | 8.39 | 0.000 | 12 | -5 | 24 | No label found |
|  | 5.08 | 0.010 | 30 | 32 | 17 | 11% Superior Longitudinal Fasciculus 3 R |
|  | 4.84 | 0.025 | -28 | -21 | 54 | 6% Corticospinal Tract L |
|  | 4.76 | 0.032 | 37 | -46 | 16 | 54% Arcuate Fasciculus R, 33% Middle Longitudinal Fasciculus R, 10% Optic Radiation R, 9% Inferior Fronto-Occipital Fasciculus R |
|  | 4.64 | 0.049 | 11 | -44 | 14 | Cingulum R |
| **Somatosensory (L)** | 12.36 | 0.000 | -31 | -19 | 32 | 59% Corticospinal Tract L, 6% Superior Longitudinal Fasciculus 2 L |
|  | 5.34 | 0.002 | -46 | -56 | 1 | No label found |
|  | 4.75 | 0.020 | -49 | -45 | -11 | 16% Inferior Longitudinal Fasciculus L |
|  | 4.55 | 0.041 | 8 | -16 | 62 | No label found |
| **Somatosensory (R)** | 13.02 | 0.000 | 37 | -26 | 37 | 8% Arcuate Fasciculus R |
|  | 4.75 | 0.009 | 48 | -34 | -11 | 93% Inferior Longitudinal Fasciculus R |
|  | 4.29 | 0.045 | 36 | 0 | 49 | No label found |
| **Speech Arrest (L)** | 37.55 | 0.000 | -21 | -7 | 25 | 13% Superior Thalamic Radiation L |
| **Speech Arrest (R)** | 17.32 | 0.000 | 22 | -7 | 26 | 15% Superior Thalamic Radiation R |
| **Verbal apraxia (L)** | 7.52 | 0.000 | -26 | -6 | 32 | 67% Superior Thalamic Radiation L |
|  | 5.12 | 0.006 | -36 | -3 | -2 | No label found |
|  | 4.90 | 0.014 | -35 | -35 | 29 | 42% Arcuate Fasciculus L, 12% Superior Longitudinal Fasciculus 2 L |
|  | 4.82 | 0.019 | -17 | -7 | 51 | 83% Superior Longitudinal Fasciculus 1 L, 78% Superior Thalamic Radiation L |
| **Verbal apraxia (R)** | 11.29 | 0.000 | 21 | -8 | 26 | 11% Superior Thalamic Radiation R |
|  | 5.47 | 0.050 | 7 | -23 | -33 | 89% Corticospinal Tract R |
| **Perseveration (L)** | 13.18 | 0.000 | -39 | -60 | 5 | 12% Inferior Fronto-Occipital Fasciculus L, 6% Middle Longitudinal Fasciculus L |
|  | 5.41 | 0.010 | -6 | -4 | 29 | 5% Cingulum subsection: Dorsal L |
| **Neologistic** | No suprathreshold clusters | | | | |  |
| **Phonology (L)** | 11.78 | 0.000 | -29 | -34 | 26 | No label found |
|  | 5.15 | 0.007 | 41 | -9 | -22 | 29% Inferior Longitudinal Fasciculus R |
|  | 5.08 | 0.009 | -12 | -3 | -7 | No label found |
|  | 4.66 | 0.041 | -9 | 8 | 22 | No label found |
| **Phonology (R)** | N/A |  |  |  |  |  |
| **Naming (L)** | 20.62 | 0.000 | -33 | -43 | 17 | 61% Middle Longitudinal Fasciculus L, 28% Optic Radiation L, 19% Inferior Fronto-Occipital Fasciculus L, 9% Acoustic Radiation L |
|  | 4.35 | 0.033 | -36 | 48 | -6 | 9% Forceps Minor |
| **Naming (R)** | No suprathreshold clusters | | | | |  |
| **Auditory Comprehension (L)** | 18.64 | 0.000 | -36 | -44 | 12 | 76% Middle Longitudinal Fasciculus L, 48% Inferior Fronto-Occipital Fasciculus L, 25% Optic Radiation L, 12% Arcuate Fasciculus L, 9% Acoustic Radiation L |
|  | 5.12 | 0.009 | -41 | 1 | 45 | No label found |
|  | 4.71 | 0.040 | -36 | -10 | -8 | 20% Inferior Fronto-Occipital Fasciculus L |
|  | 4.67 | 0.046 | -27 | 2 | -37 | 11% Fornix L |
|  | 4.67 | 0.047 | -28 | 1 | -36 | 9% Fornix L |
| **Auditory Comprehension (R)** | N/A |  |  |  |  |  |
| **Verbal Semantics (L)** | 13.91 | 0.000 | -34 | -54 | 17 | 72% Middle Longitudinal Fasciculus L, 34% Inferior Fronto-Occipital Fasciculus L, 34% Optic Radiation L |
| **Verbal Semantics (R)** | No suprathreshold clusters | | | | |  |
| **Non-verbal Semantics (L)** | 9.86 | 0.000 | -35 | -45 | 14 | 85% Middle Longitudinal Fasciculus L, 52% Inferior Fronto-Occipital Fasciculus L, 39% Optic Radiation L, 10% Arcuate Fasciculus L, 5% Acoustic Radiation L |
|  | 5.44 | 0.022 | -31 | 20 | 19 | 16% Arcuate Fasciculus L, 9% Frontal Aslant Tract L |
|  | 5.20 | 0.042 | -35 | -25 | 28 | 83% Arcuate Fasciculus L, 12% Superior Longitudinal Fasciculus 3 L |
| **Non-verbal Semantics (R)** | 7.41 | 0.000 | 38 | -50 | 12 | 24% Middle Longitudinal Fasciculus R, 5% Arcuate Fasciculus R, 5% Optic Radiation R |
|  | 6.69 | 0.001 | 32 | 15 | 19 | 62% Superior Longitudinal Fasciculus 3 R, 53% Frontal Aslant Tract R, 17% Arcuate Fasciculus R |
| **Mentalizing (L)** | Not generated | | | | |  |
| **Mentalizing (R)** | Not generated | | | | |  |
| **Reading (L)** | 10.57 | 0.000 | -24 | -33 | 26 | No label found |
|  | 4.57 | 0.041 | -26 | -83 | -3 | 11% Forceps Major, 9% Inferior Fronto-Occipital Fasciculus L, 8% Optic Radiation L, 6% Vertical Occipital Fasciculus L |
| **Reading (R)** | N/A |  |  |  |  |  |
| **Writing (L)** | 10.11 | 0.000 | -31 | -43 | 20 | 18% Middle Longitudinal Fasciculus L, 16% Optic Radiation L |
|  | 4.87 | 0.043 | -68 | -32 | 16 |  |
|  | 4.84 | 0.047 | -66 | -27 | 14 |  |
| **Writing (R)** | Not generated | | | | |  |
| **Visuospatial (L)** | Not generated | | | | |  |
| **Visuospatial (R)** | 8.59 | 0.000 | 33 | -33 | 23 | 82% Arcuate Fasciculus R |
| **Visual (L)** | No suprathreshold clusters | | | | |  |
| **Visual (R)** | No suprathreshold clusters | | | | |  |
| **Face perception (L)** | No suprathreshold clusters | | | | |  |
| **Face perception (R)** | 15.65 | 0.000 | 18 | -70 | -13 | No label found |

## **2.8 Disconnectome maps**

AF: Arcuate Fasciculus, CST: Corticospinal Tract, IC: Internal Capsule, IFOF: Inferior Fronto-Occipital Fasciculus, ILF: Inferior Longitudinal, Fasciculus, OR: Optic Radiation, SLF: Superior Longitudinal Fasciculus, UF: Uncinate Fasciculus


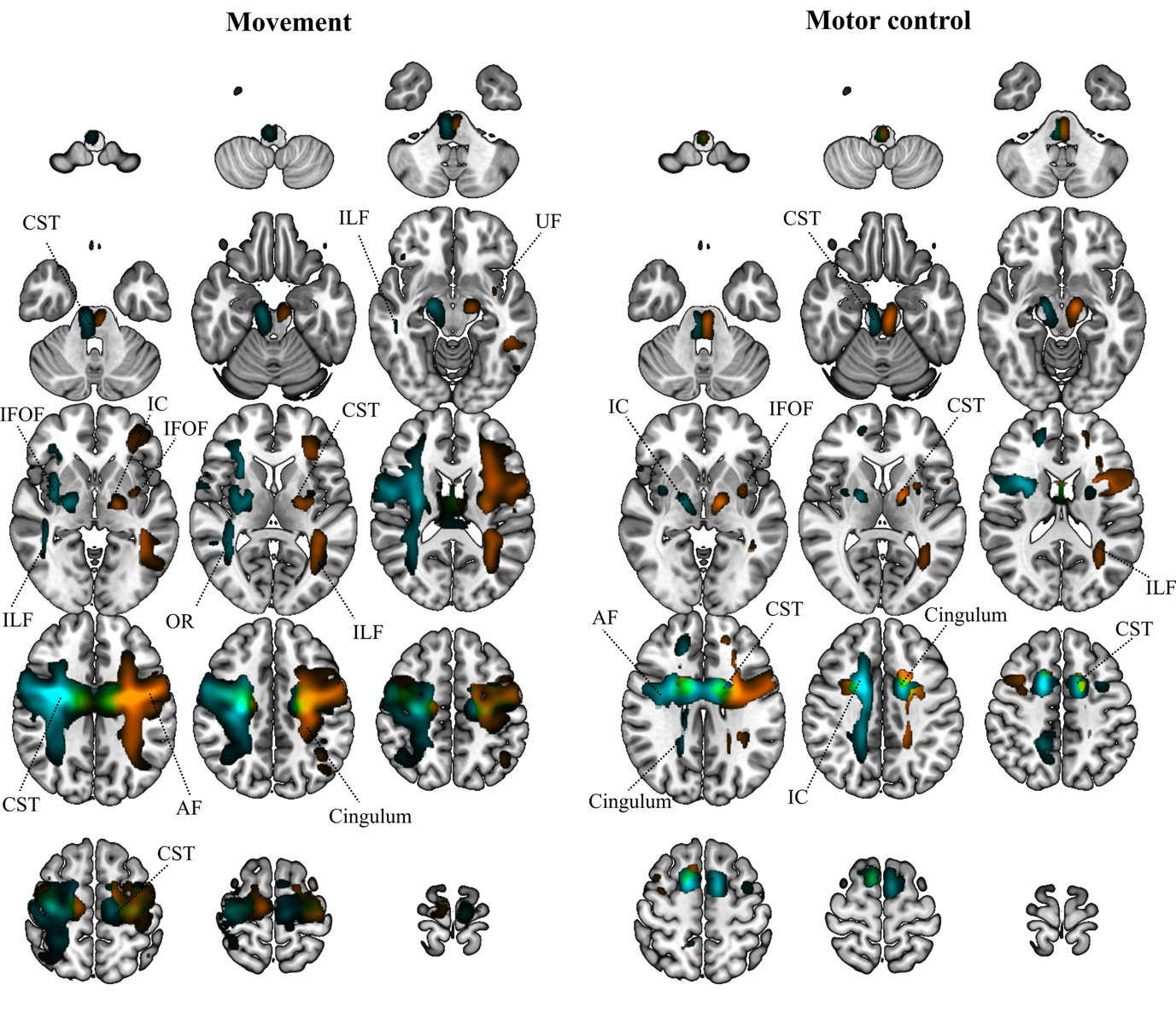


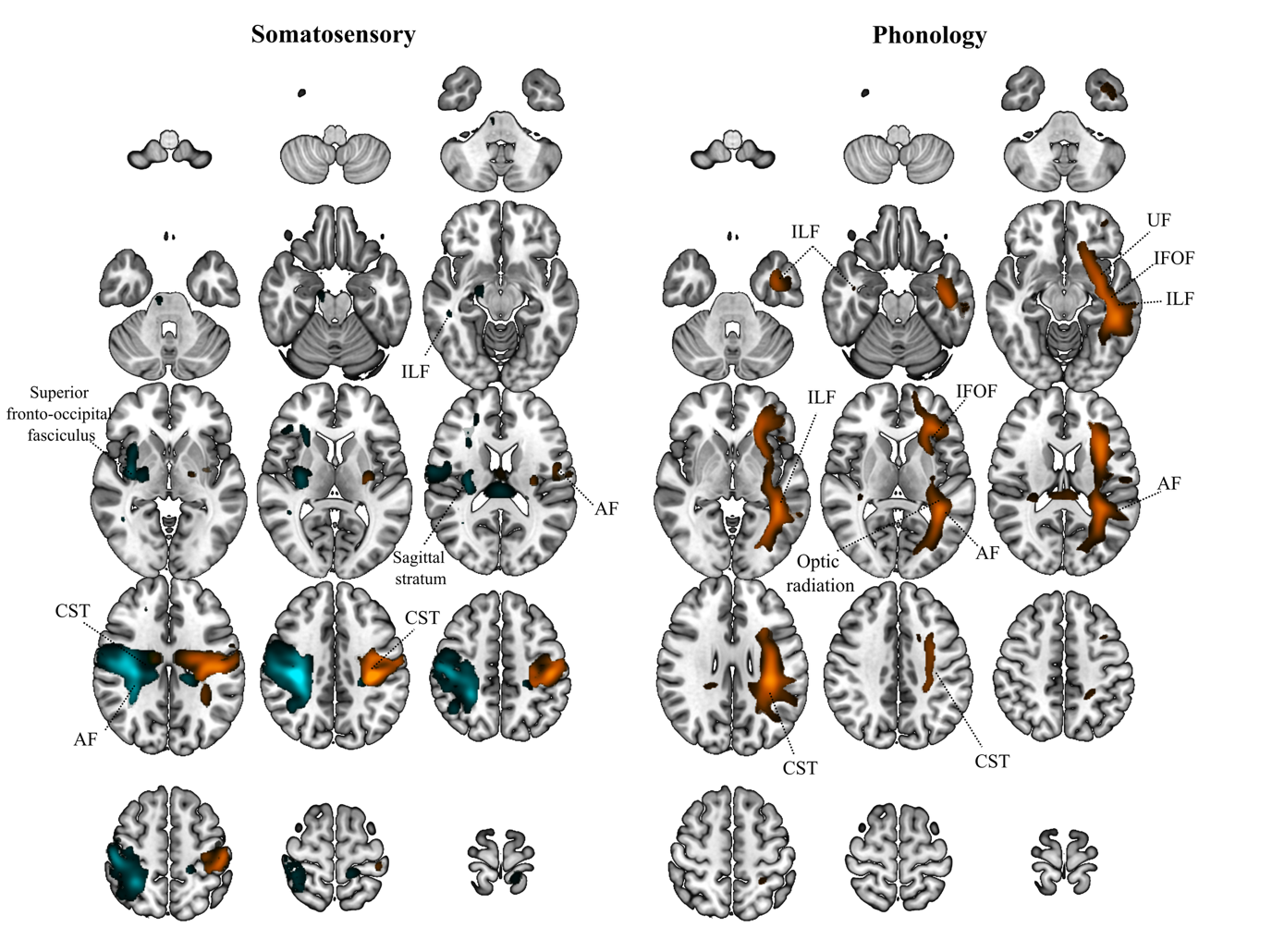


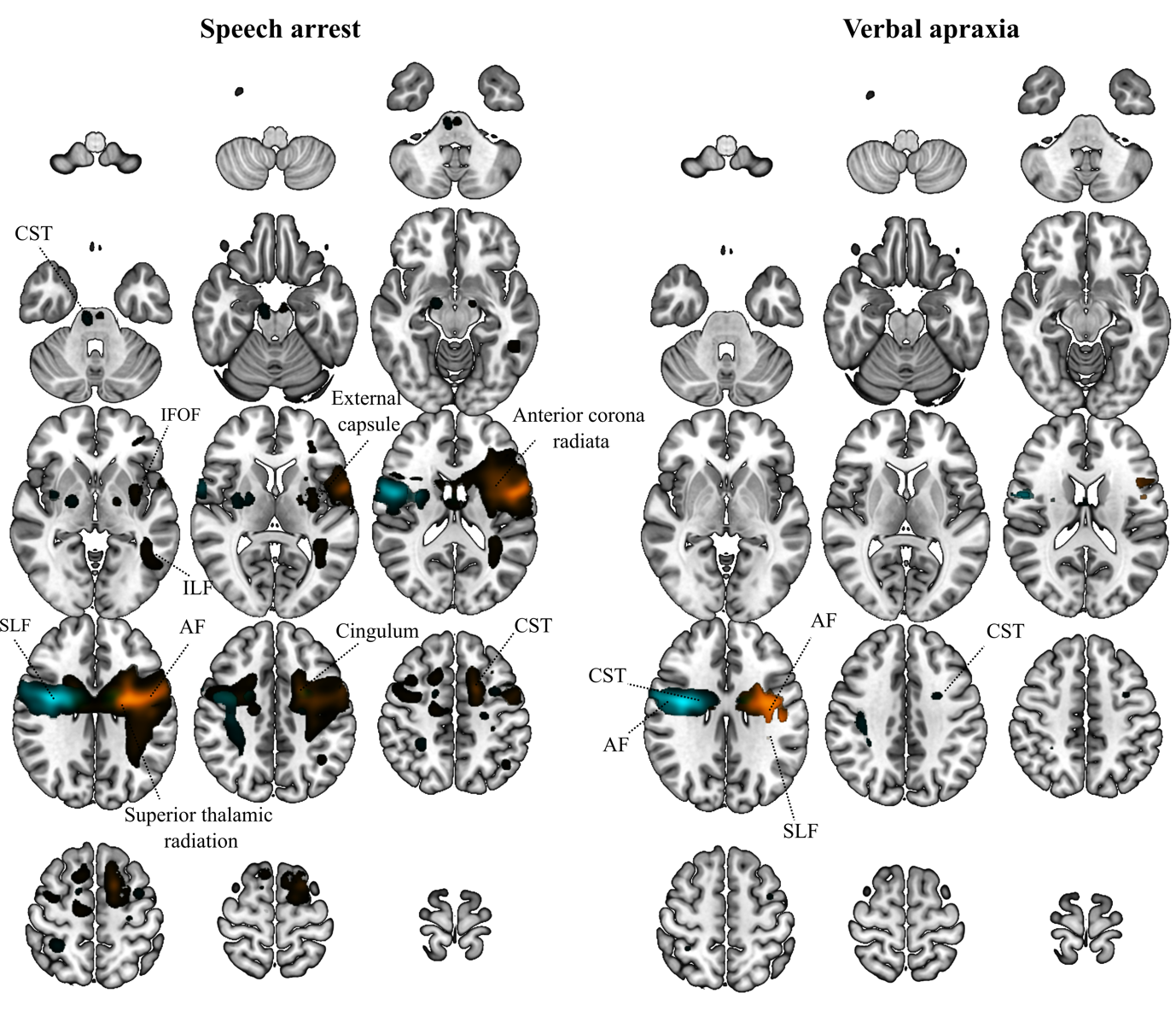


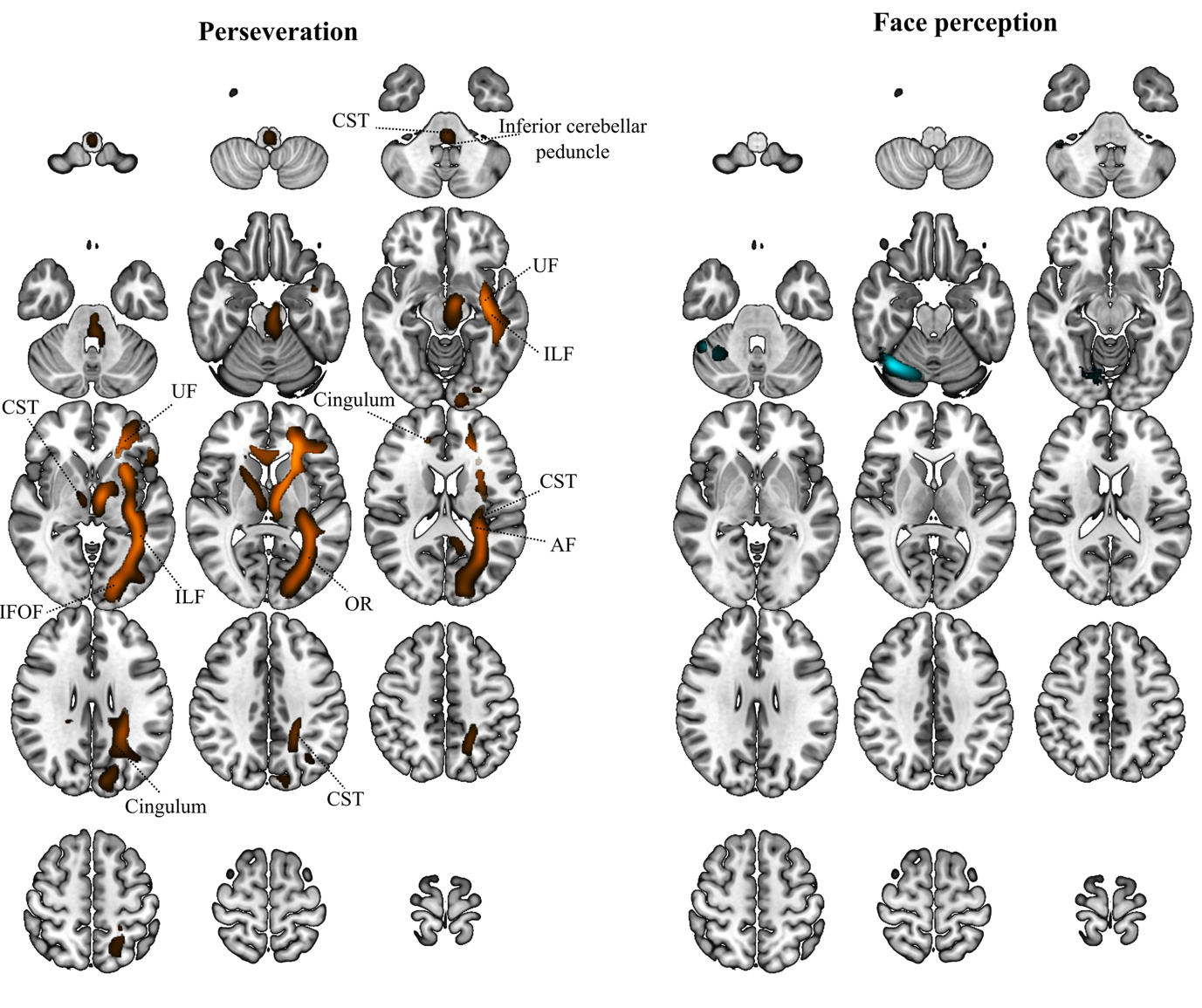


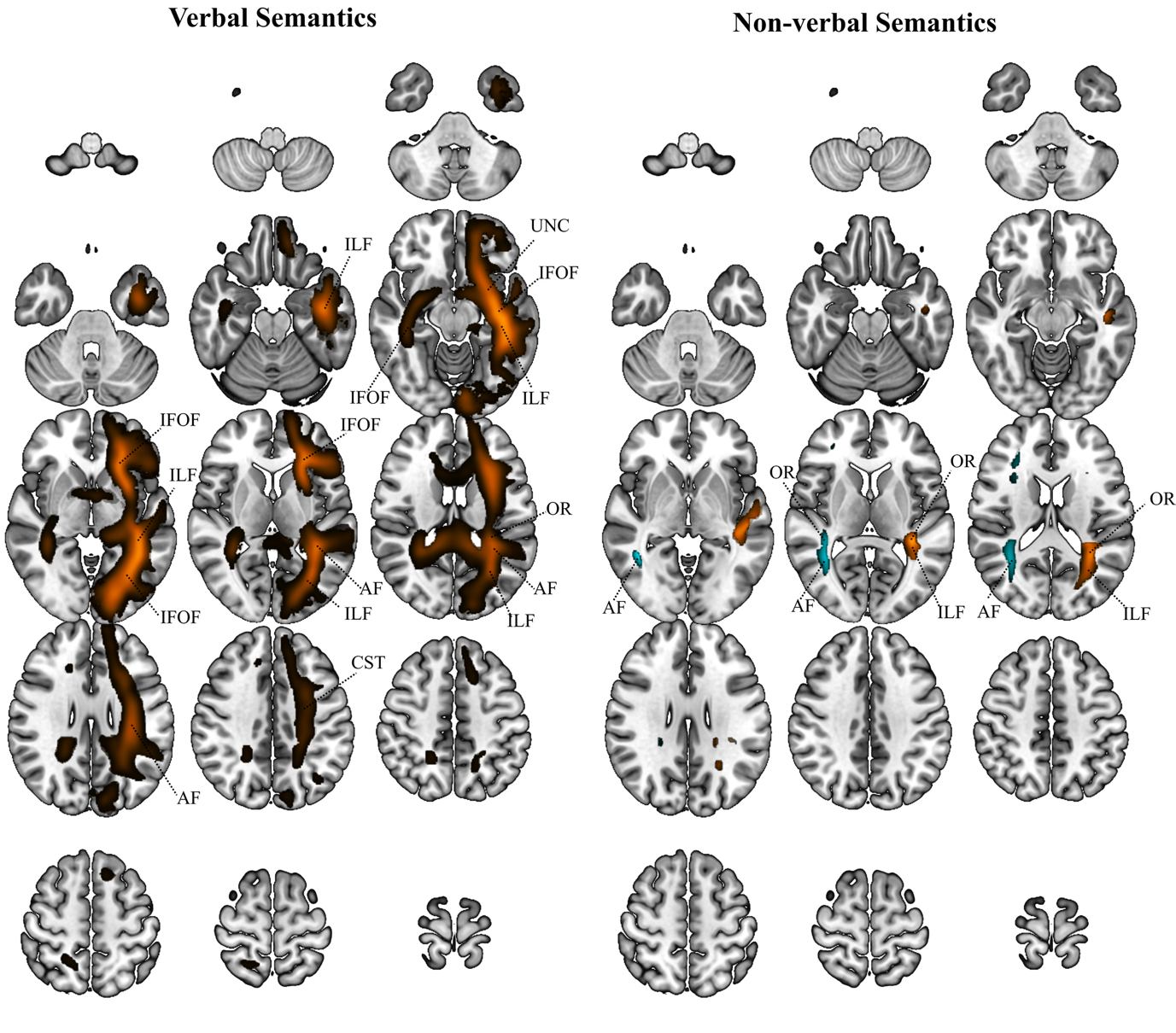


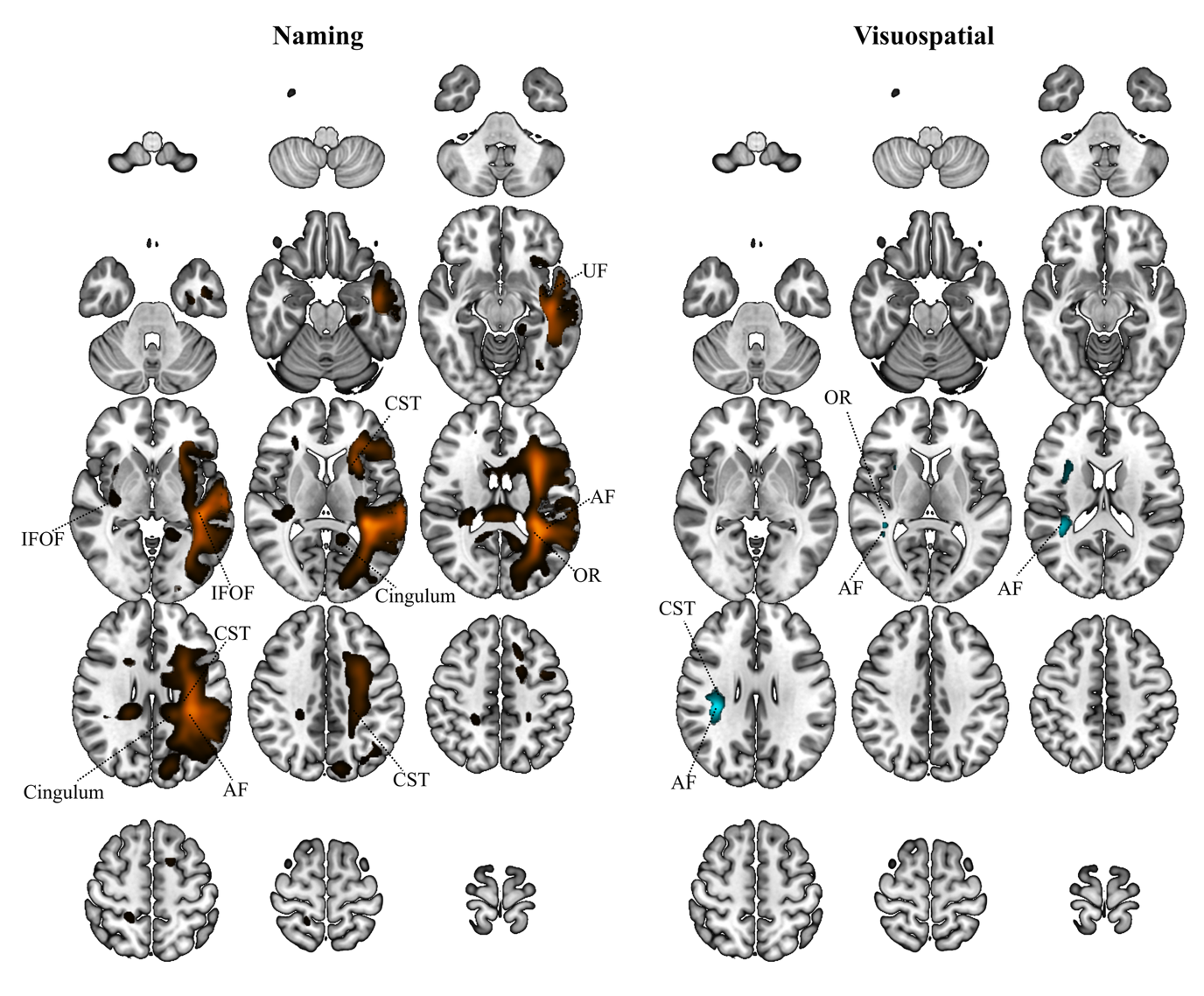


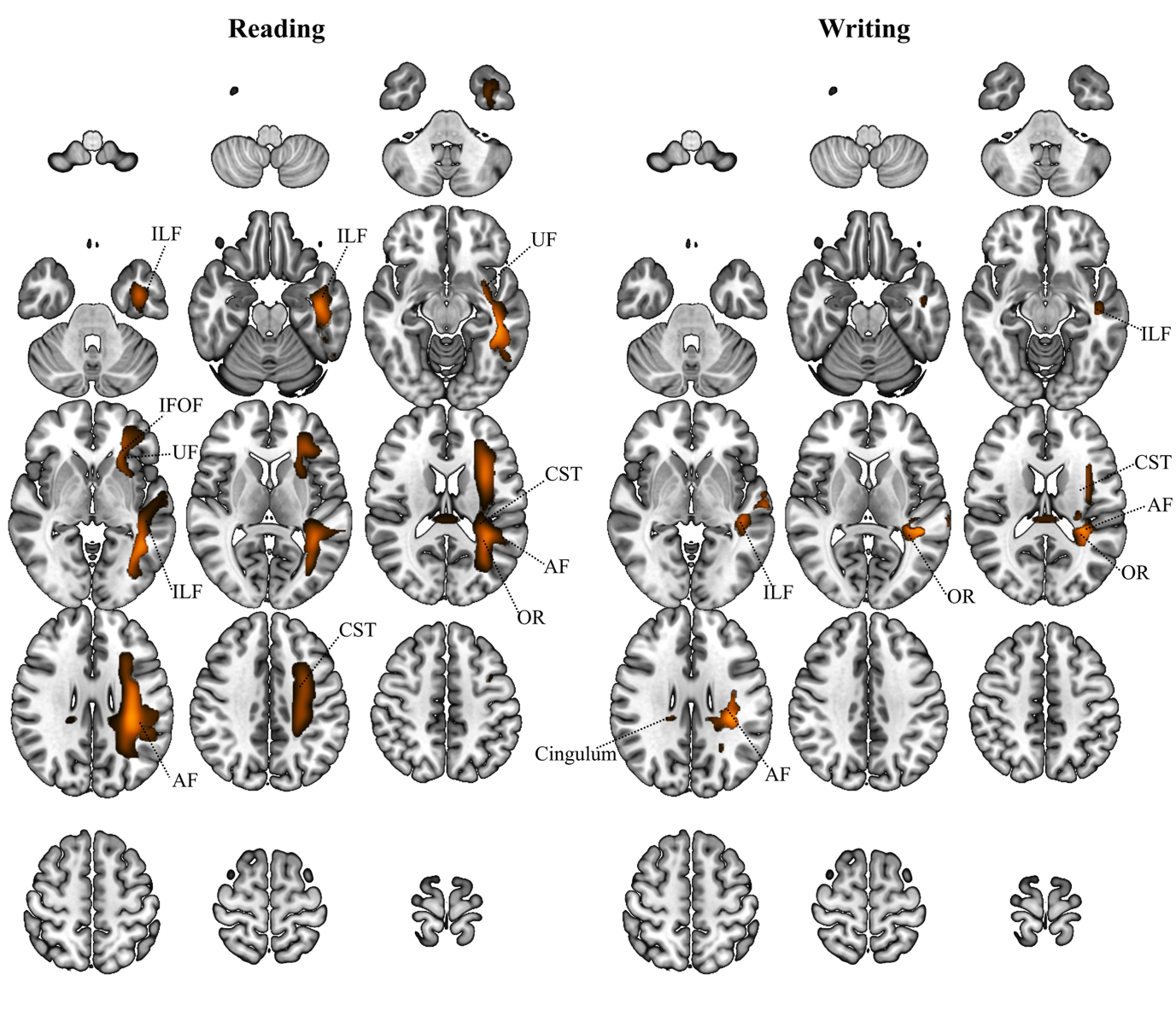


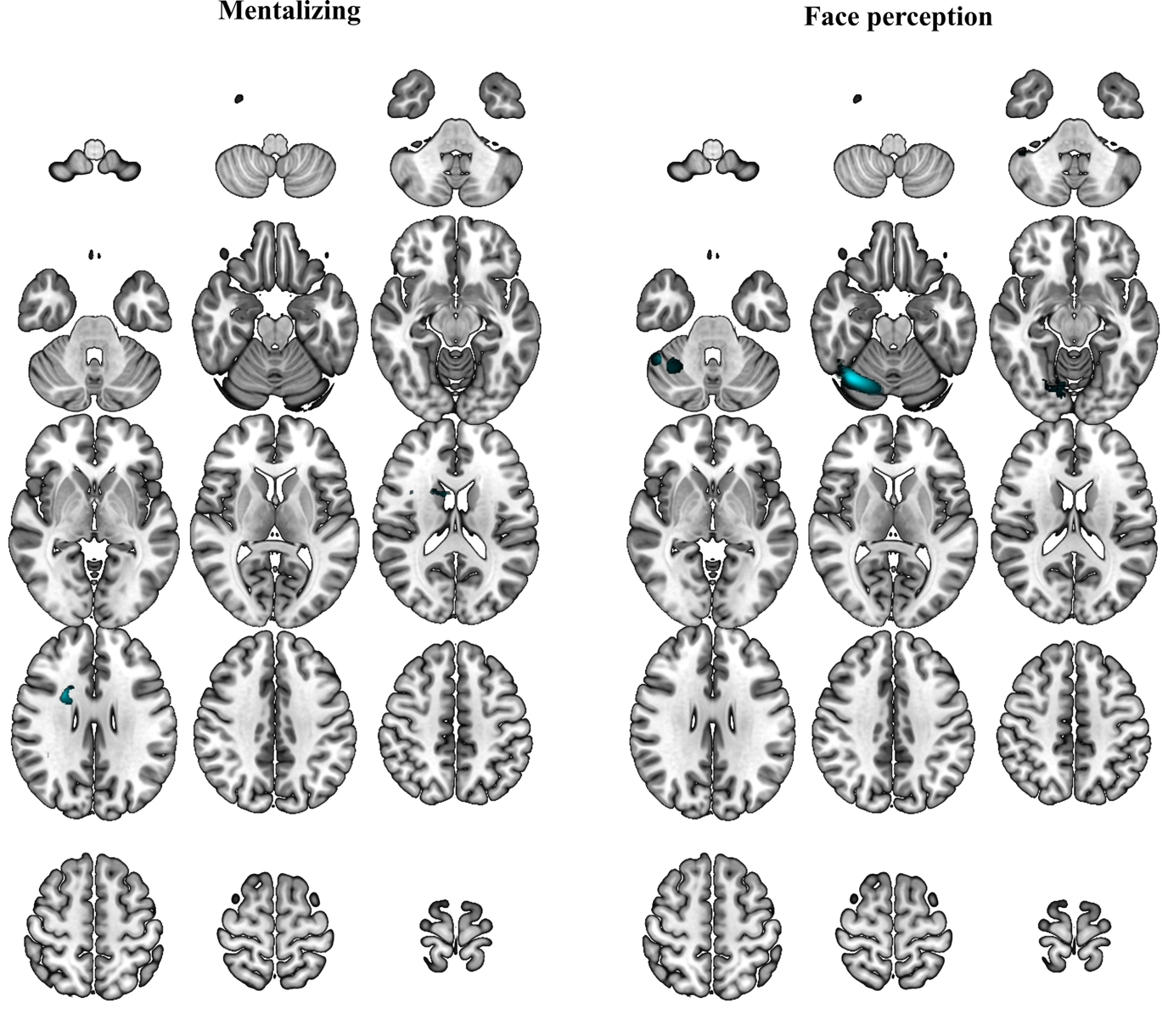


## **2.9 Maps of the distribution of stimulation points**

**
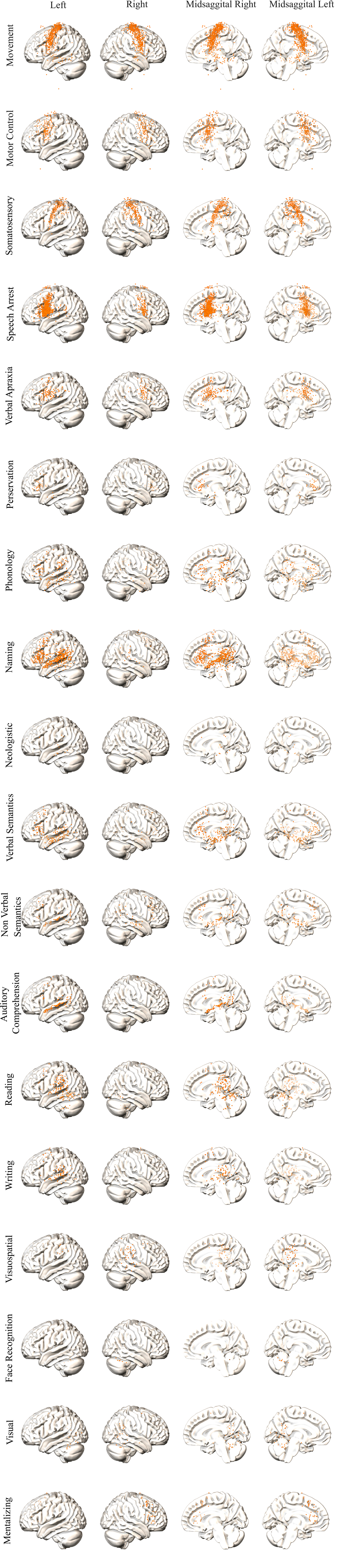
**

**
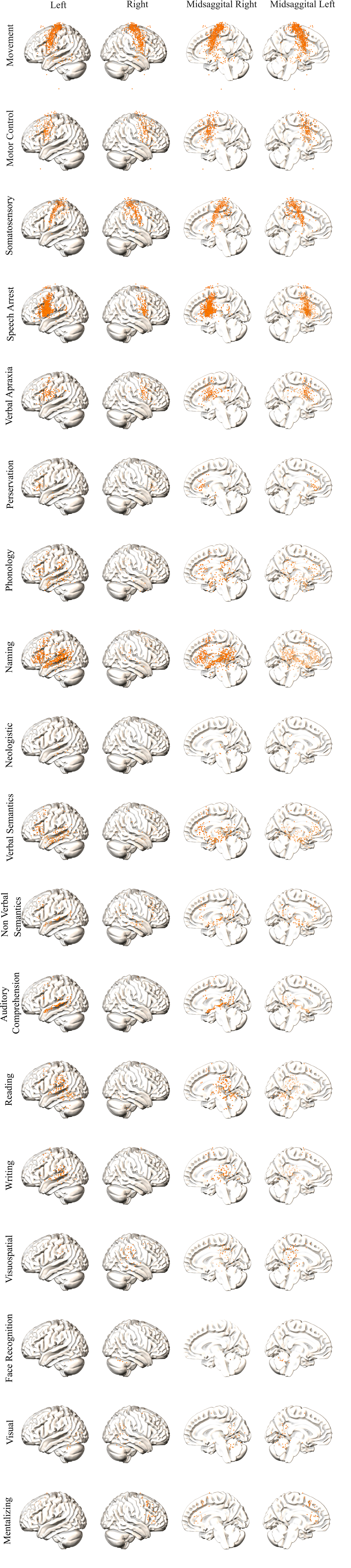
**

**
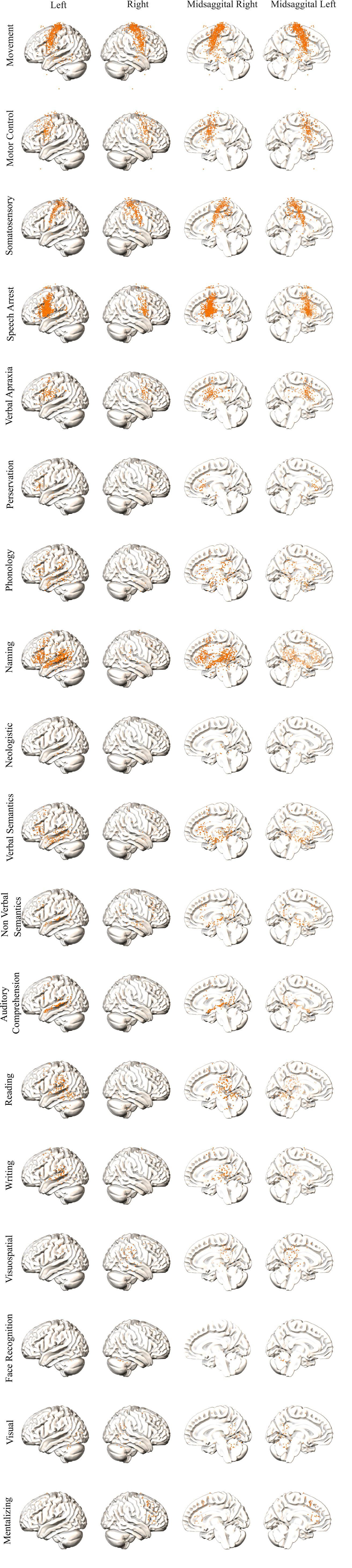
**

**REFERENCES**

Chang EF, Breshears JD, Raygor KP, Lau D, Molinaro AM, Berger MS (2017) Stereotactic probability and variability of speech arrest and anomia sites during stimulation mapping of the language dominant hemisphere. J Neurosurg 126 (1):114-121. doi:10.3171/2015.10.Jns151087

Eickhoff SB, Bzdok D, Laird AR, Kurth F, Fox PT (2012) Activation likelihood estimation meta-analysis revisited. Neuroimage 59 (3):2349-2361. doi:10.1016/j.neuroimage.2011.09.017

Eickhoff SB, Laird AR, Grefkes C, Wang LE, Zilles K, Fox PT (2009) Coordinate-based activation likelihood estimation meta-analysis of neuroimaging data: a random-effects approach based on empirical estimates of spatial uncertainty. Hum Brain Mapp 30 (9):2907-2926. doi:10.1002/hbm.20718

Foulon C, Cerliani L, Kinkingnéhun S, Levy R, Rosso C, Urbanski M, Volle E, Thiebaut de Schotten M (2018) Advanced lesion symptom mapping analyses and implementation as BCBtoolkit. Gigascience 7 (3):1-17. doi:10.1093/gigascience/giy004

Giampiccolo D, Moritz-Gasser S, Ng S, Lemaître AL, Duffau H (2022) Jargonaphasia as a disconnection syndrome: A study combining white matter electrical stimulation and disconnectome mapping. Brain Stimul 15 (1):87-95. doi:10.1016/j.brs.2021.11.012

Herbet G, Lafargue G, Moritz-Gasser S, Bonnetblanc F, Duffau H (2015) Interfering with the neural activity of mirror-related frontal areas impairs mentalistic inferences. Brain Struct Funct 220 (4):2159-2169. doi:10.1007/s00429-014-0777-x

Herbet G, Moritz-Gasser S, Boiseau M, Duvaux S, Cochereau J, Duffau H (2016) Converging evidence for a cortico-subcortical network mediating lexical retrieval. Brain 139 (11):3007-3021. doi:10.1093/brain/aww220

Herbet G, Moritz-Gasser S, Duffau H (2017a) Direct evidence for the contributive role of the right inferior fronto-occipital fasciculus in non-verbal semantic cognition. Brain Struct Funct 222 (4):1597-1610. doi:10.1007/s00429-016-1294-x

Herbet G, Yordanova YN, Duffau H (2017b) Left Spatial Neglect Evoked by Electrostimulation of the Right Inferior Fronto-occipital Fasciculus. Brain Topogr 30 (6):747-756. doi:10.1007/s10548-017-0574-y

Mandonnet E, Herbet G, Moritz-Gasser S, Poisson I, Rheault F, Duffau H (2019) Electrically induced verbal perseveration: A striatal deafferentation model. Neurology 92 (6):e613-e621. doi:10.1212/wnl.0000000000006880

Mani J, Diehl B, Piao Z, Schuele SS, Lapresto E, Liu P, Nair DR, Dinner DS, Lüders HO (2008) Evidence for a basal temporal visual language center: cortical stimulation producing pure alexia. Neurology 71 (20):1621-1627. doi:10.1212/01.wnl.0000334755.32850.f0

Matsumoto R, Imamura H, Inouchi M, Nakagawa T, Yokoyama Y, Matsuhashi M, Mikuni N, Miyamoto S, Fukuyama H, Takahashi R, Ikeda A (2011) Left anterior temporal cortex actively engages in speech perception: A direct cortical stimulation study. Neuropsychologia 49 (5):1350-1354. doi:10.1016/j.neuropsychologia.2011.01.023

Mori S, Wakana S, Van Zijl PC, Nagae-Poetscher LM (2005) MRI atlas of human white matter. 1 edn. Elsevier,

Ng S, Moritz-Gasser S, Lemaitre AL, Duffau H, Herbet G (2021) White matter disconnectivity fingerprints causally linked to dissociated forms of alexia. Commun Biol 4 (1):1413. doi:10.1038/s42003-021-02943-z

Page MJ, McKenzie JE, Bossuyt PM, Boutron I, Hoffmann TC, Mulrow CD, Shamseer L, Tetzlaff JM, Akl EA, Brennan SE, Chou R, Glanville J, Grimshaw JM, Hróbjartsson A, Lalu MM, Li T, Loder EW, Mayo-Wilson E, McDonald S, McGuinness LA, Stewart LA, Thomas J, Tricco AC, Welch VA, Whiting P, Moher D (2021) The PRISMA 2020 statement: an updated guideline for reporting systematic reviews. Bmj 372:n71. doi:10.1136/bmj.n71

Rech F, Herbet G, Moritz-Gasser S, Duffau H (2016) Somatotopic organization of the white matter tracts underpinning motor control in humans: an electrical stimulation study. Brain Struct Funct 221 (7):3743-3753. doi:10.1007/s00429-015-1129-1

Roux FE, Dufor O, Giussani C, Wamain Y, Draper L, Longcamp M, Démonet JF (2009) The graphemic/motor frontal area Exner's area revisited. Ann Neurol 66 (4):537-545. doi:10.1002/ana.21804

Roux FE, Durand JB, Jucla M, Réhault E, Reddy M, Démonet JF (2012) Segregation of lexical and sub-lexical reading processes in the left perisylvian cortex. PLoS One 7 (11):e50665. doi:10.1371/journal.pone.0050665

Roux FE, Durand JB, Réhault E, Planton S, Draper L, Démonet JF (2014) The neural basis for writing from dictation in the temporoparietal cortex. Cortex 50:64-75. doi:10.1016/j.cortex.2013.09.012

Roux FE, Miskin K, Durand JB, Sacko O, Réhault E, Tanova R, Démonet JF (2015) Electrostimulation mapping of comprehension of auditory and visual words. Cortex 71:398-408. doi:10.1016/j.cortex.2015.07.001

Sarubbo S, Tate M, De Benedictis A, Merler S, Moritz-Gasser S, Herbet G, Duffau H (2020) Mapping critical cortical hubs and white matter pathways by direct electrical stimulation: an original functional atlas of the human brain. Neuroimage 205:116237. doi:10.1016/j.neuroimage.2019.116237

Schrouff J, Raccah O, Baek S, Rangarajan V, Salehi S, Mourão-Miranda J, Helili Z, Daitch AL, Parvizi J (2020) Fast temporal dynamics and causal relevance of face processing in the human temporal cortex. Nat Commun 11 (1):656. doi:10.1038/s41467-020-14432-8

Simone L, Viganò L, Fornia L, Howells H, Leonetti A, Puglisi G, Bellacicca A, Bello L, Cerri G (2021) Distinct Functional and Structural Connectivity of the Human Hand-Knob Supported by Intraoperative Findings. J Neurosci 41 (19):4223-4233. doi:10.1523/jneurosci.1574-20.2021

Tate MC, Herbet G, Moritz-Gasser S, Tate JE, Duffau H (2014) Probabilistic map of critical functional regions of the human cerebral cortex: Broca's area revisited. Brain 137 (Pt 10):2773-2782. doi:10.1093/brain/awu168

Yordanova YN, Cochereau J, Duffau H, Herbet G (2019) Combining resting state functional MRI with intraoperative cortical stimulation to map the mentalizing network. Neuroimage 186:628-636. doi:10.1016/j.neuroimage.2018.11.046

Zacà D, Corsini F, Rozzanigo U, Dallabona M, Avesani P, Annicchiarico L, Zigiotto L, Faraca G, Chioffi F, Jovicich J, Sarubbo S (2018) Whole-Brain Network Connectivity Underlying the Human Speech Articulation as Emerged Integrating Direct Electric Stimulation, Resting State fMRI and Tractography. Front Hum Neurosci 12:405. doi:10.3389/fnhum.2018.00405

Zhou Y, Zhao Z, Zhang J, Hameed NUF, Zhu F, Feng R, Zhang X, Lu J, Wu J (2021) Electrical stimulation-induced speech-related negative motor responses in the lateral frontal cortex. J Neurosurg:1-9. doi:10.3171/2021.9.Jns211069
